# Dentinogenic effects of extracted dentin matrix components digested with matrix metalloproteinases

**DOI:** 10.1038/s41598-018-29112-3

**Published:** 2018-07-16

**Authors:** Motoki Okamoto, Yusuke Takahashi, Shungo Komichi, Paul R. Cooper, Mikako Hayashi

**Affiliations:** 1 Department of Restorative Dentistry and Endodontology, Osaka University Graduate School of Dentistry, Osaka, Japan; 2 Oral Biology, School of Dentistry, University of Birmingham, Birmingham, UK

**Keywords:** Mechanisms of disease, Dental caries

## Abstract

Dentin is primarily composed of hydroxyapatite crystals within a rich organic matrix. The organic matrix comprises collagenous structural components, within which a variety of bioactive molecules are sequestered. During caries progression, dentin is degraded by acids and enzymes derived from various sources, which can release bioactive molecules with potential reparative activity towards the dentin-pulp complex. While these molecules’ repair activities in other tissues are already known, their biological effects are unclear in relation to degradation events during disease in the dentin-pulp complex. This study was undertaken to investigate the effects of dentin matrix components (DMCs) that are partially digested by matrix metalloproteinases (MMPs) *in vitro* and *in vivo* during wound healing of the dentin-pulp complex. DMCs were initially isolated from healthy dentin and treated with recombinant MMPs. Subsequently, their effects on the behaviour of primary pulp cells were investigated *in vitro* and *in vivo*. Digested DMCs modulated a range of pulp cell functions *in vitro*. In addition, DMCs partially digested with MMP-20 stimulated tertiary dentin formation *in vivo*, which exhibited a more regular tubular structure than that induced by treatment with other MMPs. Our results indicate that MMP-20 may be especially effective in stimulating wound healing of the dentin-pulp complex.

## Introduction

When a tooth is damaged by caries and/or fracture, underlying pulp tissue can become exposed. In this situation, direct pulp capping is the only treatment option that enables preservation of pulp vitality and activation of dentin regeneration^1^. Pulp capping involves the placement of a protective dressing over the exposed pulp area to stimulate tertiary dentin formation. Calcium hydroxide [Ca(OH)_2_] has long been considered the gold standard of pulp capping materials; more recently, mineral trioxide aggregate (MTA) has found wide clinical use for this application^2,3^. Although these materials exhibit relatively high success rates (60%–80%)^4^, neither material was originally developed to induce cellular repair of the dentin-pulp complex; such treatment should be based on the key biological events associated with tissue repair. Our understanding of tertiary dentinogenesis, as well as our ability to modify this process within the clinical setting, remain incomplete; this constrains the development of optimal pulp capping materials that are compatible with the biological events occurring during dentin-pulp repair and regeneration^5–7^.

Therefore, we aimed to determine how MMPs may influence tertiary dentinogenic events to support the development of a new generation of biologically-based pulp capping materials. We previously reported that matrix metalloproteinases (MMPs) are involved in wound healing within the dentin-pulp complex^8^. Notably, the integrity of the extracellular matrix (ECM) of many organs can be affected by MMPs and tissue inhibitor of metalloproteinases (TIMPs), both of which play critical roles in matrix remodeling^9–11^ by regulating ECM digestion^12,13^. Indeed, when ECM from heart, kidney, skin, bladder, or skin tissues were digested enzymatically or treated with acid, they released ECM molecules and molecular or peptide fragments, including growth factors and other signalling molecules reported to significantly enhance tissue repair-associated cellular activities^14–16^. During caries the dissolution of dentin matrix by bacterial acids and hydrolytic enzymes leads to the release of dentin matrix components (DMCs), as well as partially digested molecular components^17^. In addition, several MMPs are reported to be constituents of the dentin-pulp complex^17–20^; MMPs can further induce the activity of bacterial enzymes, which may result in activation of latent hydrolytic enzymes. Subsequently, the combined effects of activated bacterial enzymes and MMPs, specifically regarding the partial cleavage of molecules within the dentin matrix, could influence signalling aspects of tertiary dentinogenesis. This supports the concept that bioactive molecules within the dentin matrix can exert signalling properties when not intact.

While bacterial and host enzymes are important for caries progression^21^, their exact role in hard tissue formation in the dentin-pulp complex remains unclear. Additionally, it is unclear whether partial digestion of DMCs impacts their bioactive properties and their effects on wound healing in the dentin-pulp complex. Therefore, we hypothesize that DMCs digested with MMPs can enhance tertiary dentinogenic events in the dentin-pulp complex. In this study, we investigated the effects of partially MMP-digested DMCs on pulp cell functions related to wound healing *in vitro* and wound healing in the dentin-pulp complex *in vivo*.

## Results

### Protein profile of digested DMCs

**Figure 1:**
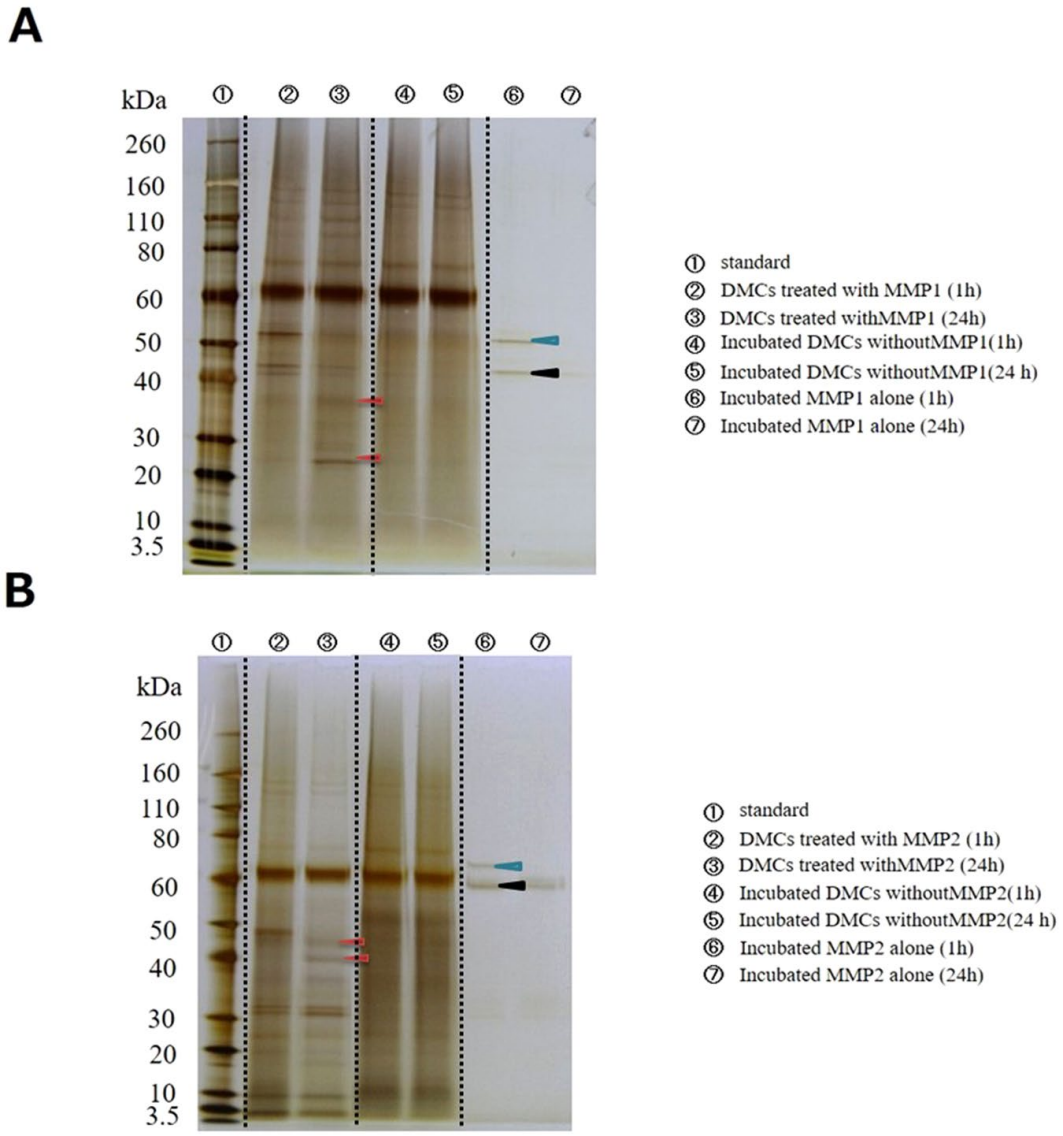
Protein profiles (silver staining) of dentin matrix components (DMCs) treated with matrix metalloproteinase (MMP)-1 (**A**) or MMP-2 (**B**) are shown as a merged view by combining images of the cropped blots and gels. Dotted lines indicate the borders of the cropped blots. The incubation time influenced the protein profile. Red arrowheads indicate the differences between protein profiles of MMP-treated DMCs following 1- and 24-h incubation. Light blue arrowhead indicates pro form of predicted MMP-1 and MMP-2, respectively. Black arrowhead indicates active form of predicted MMP-1 and MMP-2. Original full blots/gels are presented in Supplementary Fig. 13.

Sodium dodecyl sulfate-polyacrylamide gel electrophoresis (SDS-PAGE) showed that DMCs were partially digested by MMP-1, -2, -3, -8, -9, -13, or -20 after 1- and 24-h incubation, compared with undigested DMCs; longer incubation periods resulted in greater digestion. Representative protein profiles following MMP-1 and −2 treatments for 1 and 24 h are shown in Fig. 1A,B (red arrowheads), respectively. DMC samples incubated in the absence of MMPs for the same period of time serve as controls for the effects of any endogenous enzyme activities within the DMC preparations. In subsequent experiments, DMCs treated with MMPs for 24 h were analysed to enable the study of the effects of long-term digestion. Other MMPs also showed distinctive digestion profiles (Supplementary Figs 1–3).

### Angiogenesis assay

**Figure 2:**
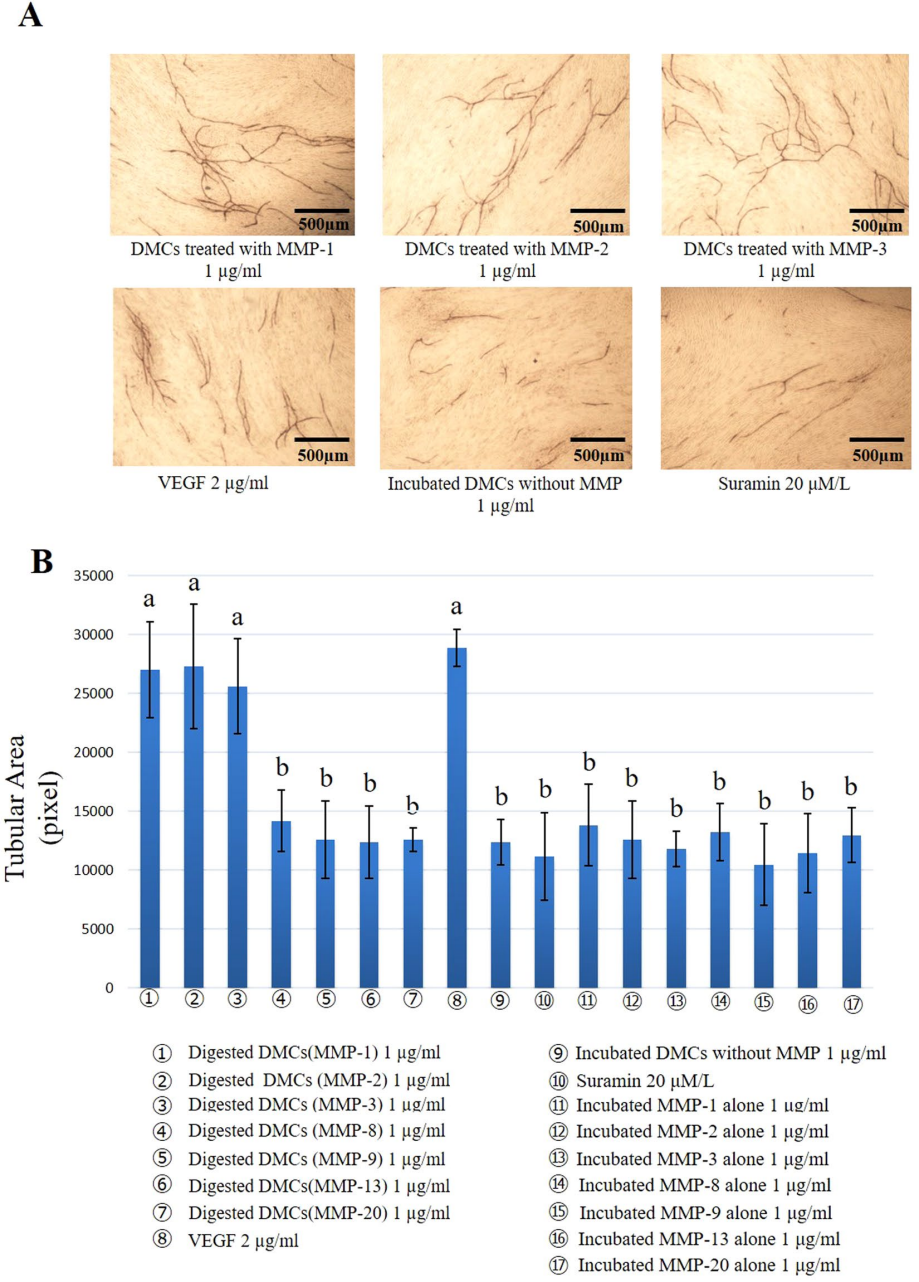
*In vitro* angiogenesis assay. (**A**) Representative CD31 immunostaining image after 11-day culture in the presence of 1 μg/ml of dentin matrix components (DMCs) treated with matrix metalloproteinases (MMP)-1, -2 and -3, incubated DMCs without MMP were used, 20 µM/L suramin as a negative control or 2 ng/ml of vascular endothelial growth factor (VEGF) as a positive control (×40). (**B**) Quantification of tubule formation. One microgram per millilitre of DMCs treated with MMP-1, -2, and -3 significantly promoted angiogenesis, compared with the negative control, DMCs incubated without MMP and MMPs incubated alone (p < 0.05). No significant differences were observed with 1 µg/ml of DMCs treated with the other MMPs, MMPs incubated alone or at lower concentrations (0.01–0.1 µg/ml) of the above-described MMPs. Groups with similar lower-case letters (i.e., a and b) are not significantly different. Data represent three independent experiments.

Angiogenesis was evaluated in terms of tubular area, including analysis of three parameters: vessel number, branch length, and branch point. One microgram per millilitre of DMCs digested with MMP-1, -2, or -3 promoted tubule formation, compared with 1 µg/ml of incubated DMCs without MMP digestion. Suramin was used as a negative control and vascular endothelial growth factor (VEGF) was applied as a positive control (Fig. 2A,B). No significant differences were observed between DMCs treated with MMP-8, -9, -13, or -20 and the experimental control, regardless of the concentration applied. Additionally, lower concentrations of DMCs (0.01–0.1 µg/ml) treated with MMP-1, -2, or -3 resulted in similar levels of tubule formation, compared with experimental controls (Supplementary Fig. 4). However, 1 µg/ml of DMCs digested by MMP-1, -2, or -3 showed significantly increased tubule formation, compared with experimental controls (p < 0.05) (Fig. 2B). Each MMP molecule incubated alone (0.01–1 µg/ml) was used as an additional control and showed no effect in the angiogenesis assay.

### Migration assay

**Figure 3:**
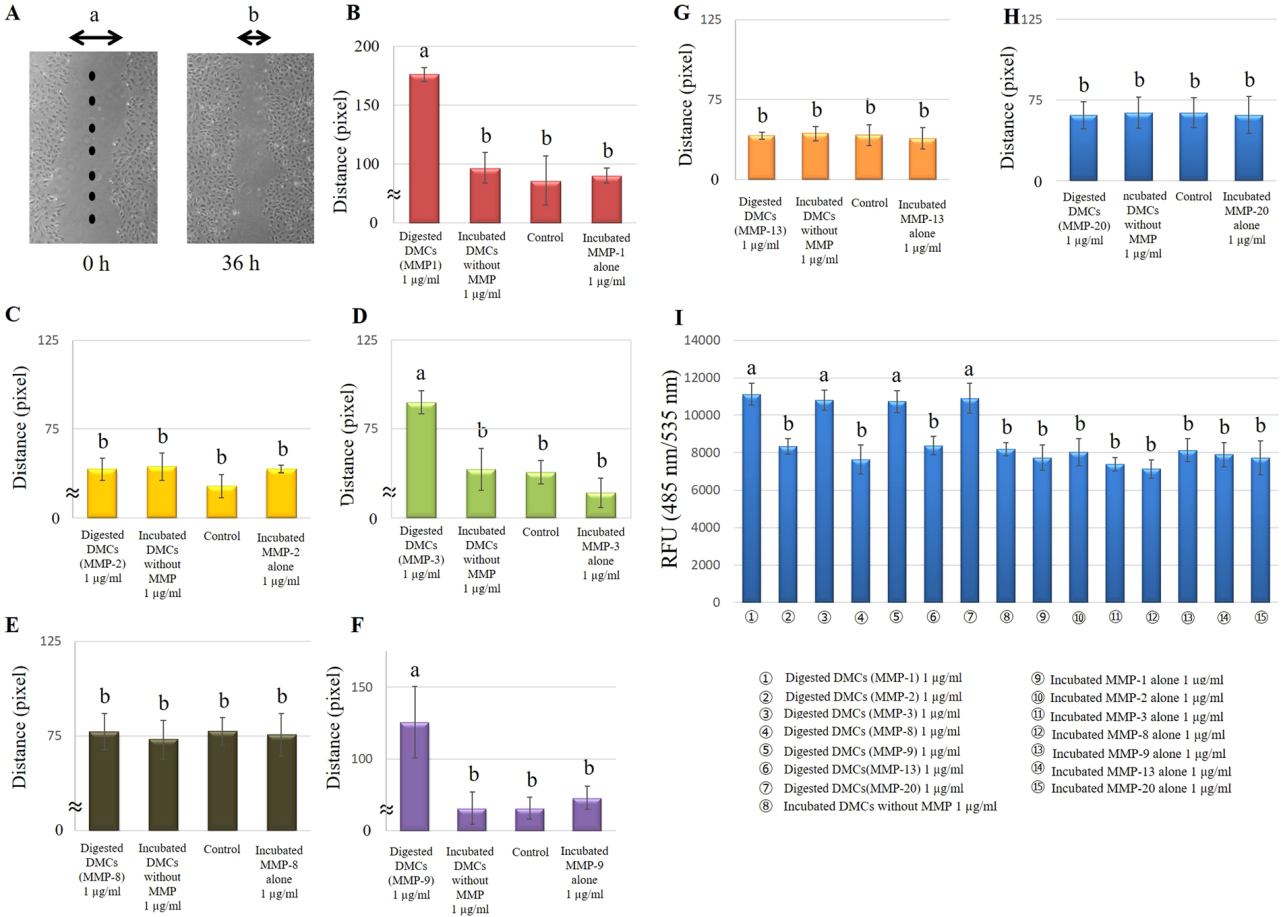
Effects of 1 µg/ml of dentin matrix components (DMCs) treated with matrix metalloproteinases (MMPs) on wound scratch (**B–H**) and chemotactic (**I**) cell migration. Eight points were randomly selected to assess wound scratch migration; the distances of the cells from the edge of the scratch wound were measured by microscopic observation (**A**). Wound scratch migration of cells incubated with 1 µg/ml DMCs treated with MMP-1 (**B**), MMP-2 (**C**), MMP-3 (**D**), MMP-8 (**E**), MMP-9 (**F**), MMP-13 (**G**) and MMP-20 (**H**) is presented as the amount of movement, and chemotactic migration of cells incubated with 1 µg/ml DMCs treated with MMP-1, MMP-2, -3, -8, -9, -13, and -20 is presented as relative fluorescence units (RFU) (I). One microgram per millilitre of DMCs treated with MMP-1, -3, or -9 promoted horizontal migration, compared with 1 µg/ml of incubated DMCs without the addition of MMPs (p < 0.05). The trans-well assay demonstrated the significant chemotactic effects of 1 µg/ml of DMCs treated with MMP-1, -3, -9, or -20, compared with the experimental and negative controls (p < 0.05). Significant differences were not observed with 1 µg/ml DMCs treated with other MMPs, MMPs incubated alone or at lower concentrations (0.01–0.1 µg/ml) of the above-mentioned MMPs. Groups with similar lower-case letters (i.e., a and b) are not significantly different. Data represent five independent experiments.

Pulp cell migration plays a critical role in repair and regeneration^22^. In the present study, two different experimental approaches were used to study migration. The horizontal scratch wound assay allowed direct visualization of the effects of 1 µg/ml of DMCs treated with MMP-1, -3, or -9 on promotion of cell migration; there was a significant difference, compared with 1 µg/ml of incubated DMCs without the addition of MMPs (p < 0.05) (Fig. 3B,D,F). Control medium (α-minimum essential medium (MEM) containing 1% fetal bovine serum (FBS)) was used as a negative control. Trans-well assays showed significant chemotactic effects of 1 µg/ml of DMCs treated with MMP-1, -3, -9, or -20, compared with an experimental control and negative control (p < 0.05) (Fig. 3E). No significant differences were observed in 1 µg/ml of DMCs digested by MMP-2, -8 or -13 samples, nor were differences observed at lower concentrations (0.01–0.1 µg/ml) of MMP-treated DMCs (Supplementary Fig. 5). Each MMP molecule incubated alone (0.01–1 µg/ml) was used as an additional control and showed no effect in the migration assay.

### Proliferation assay

**Figure 4:**
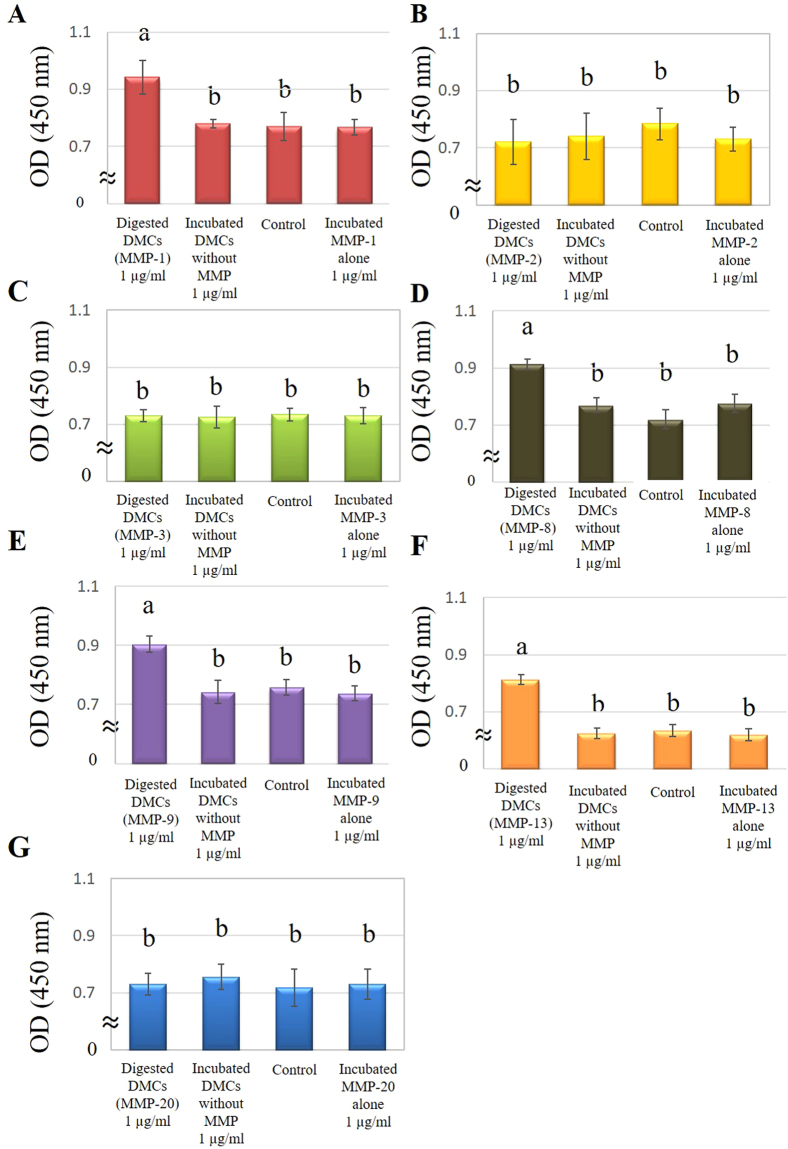
Effects of 1 µg/ml of dentin matrix components (DMCs) treated with matrix metalloproteinase (MMP)-1 (**A**), MMP-2 (**B**), MMP-3 (**C**), MMP-8 (**D**), MMP-9 (**E**), MMP-13 (**F**) and MMP-20 (**G**) on cell proliferation of rat primary pulp cells. Pulp cell proliferation was promoted by 1 µg/ml of DMCs treated with MMP-1 (**A**), -8 (**D**), -9 (**E**), or -13 (**F**), compared with incubated DMCs without the addition of MMPs (p < 0.05). Significant differences were not observed with 1 µg/ml DMCs treated with other MMPs, MMPs incubated alone or at lower concentrations (0.01–0.1 µg/ml) of the above mentioned MMPs (Supplementary Figure 6). Groups with similar lower-case letters (i.e., a and b) are not significantly different. Data represent five independent experiments.

Pulp cell proliferation was promoted by 1 µg/ml of DMCs treated with MMP-1, -8, -9, or -13, compared with DMCs incubated without MMPs (experimental control) and α-MEM containing 1% FBS (negative control) (p < 0.05) (Fig. 4). However, 1 µg/ml of DMCs treated with MMPs-2, -3, or -20, as well as lower concentrations (0.01–0.1 µg/ml) of MMP-treated DMCs, did not significantly promote cell proliferation (Supplementary Fig. 6). Each MMP molecule incubated alone (0.01–1 µg/ml) was used as an additional control and showed no effect in the proliferation assay.

### ALP activity

**Figure 5:**
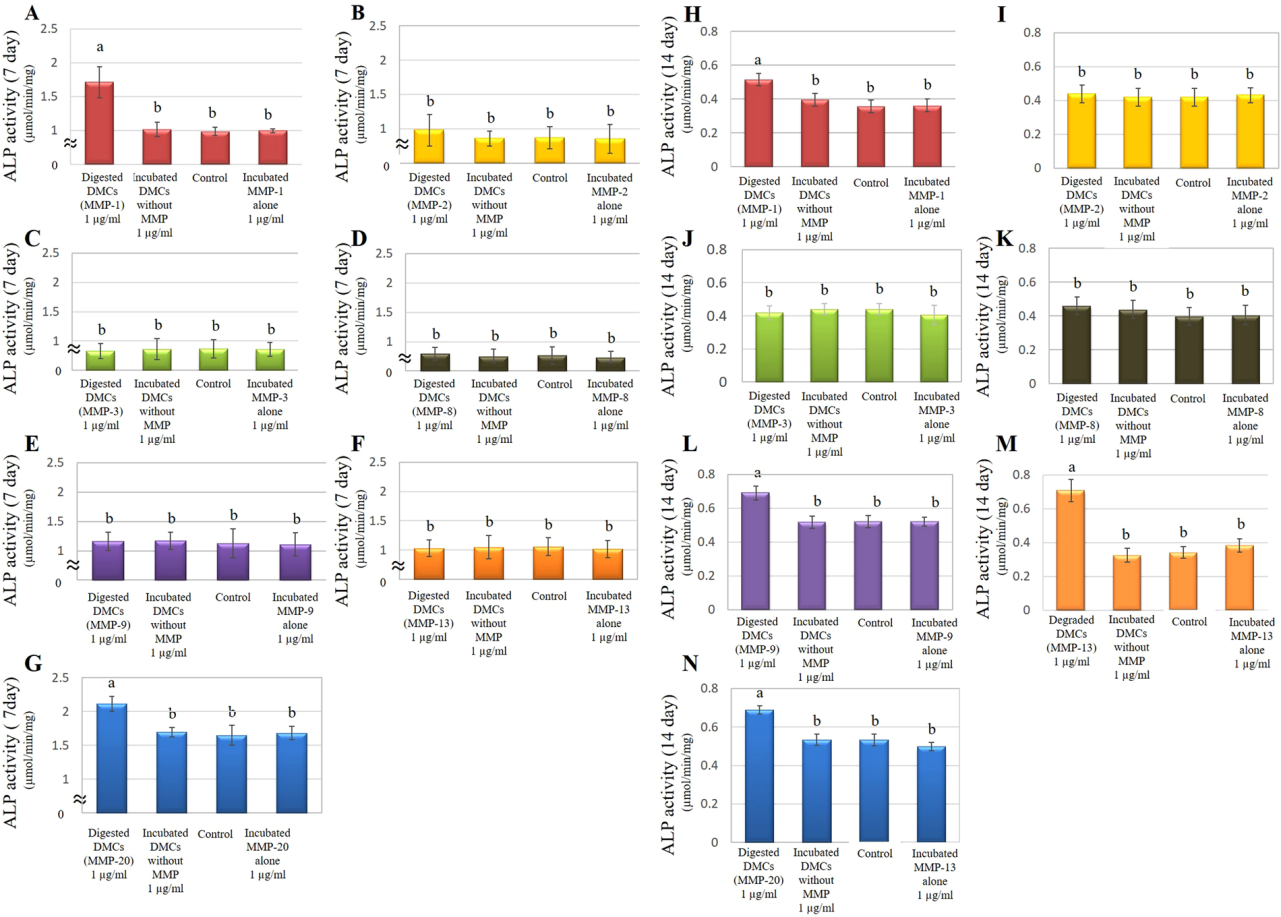
Effects of 1 µg/ml of MMP-treated dentin matrix components (DMCs) on cell differentiation in rat primary pulp cells. Cells were treated with DMCs treated with matrix metalloproteinase (MMP)-1 (**A**,**H**), MMP-2 (**B**,**I**), MMP-3 (**C,J**), MMP-8 (**D,K**), MMP-9 (**D,L**), MMP-13 (**E,M**), and MMP-20 (**G,N**), incubated DMCs without MMP or medium alone containing 50 μg/ml of ascorbic acid and 10 mM of β-glycerophosphate for 7 days (**A–G**) or 14 days (**H–N**). One microgram per millilitre of DMCs treated with MMP-1 (**A**) or MMP-20 (**G**) showed higher alkaline phosphatase (ALP) activities, compared with incubated DMCs without MMPs at 7 days (p < 0.05). On day 14, DMCs treated with MMP-9 (**L**) and -13 (**M**) also stimulated pulp cell ALP activity in addition to MMP-1 and -20 (p < 0.05). Significant differences were not observed with 1 µg/ml of DMCs treated with other MMPs, MMPs incubated alone, or at lower concentrations (0.01–0.1 µg/ml) of the above-mentioned MMPs. Groups with similar lower-case letters (i.e., a and b) are not significantly different (Supplementary Figure 7). Data represent five independent experiments.

On day 7, primary pulp cells incubated with 1 µg/ml of DMCs treated with MMP-1 or MMP-20 showed higher alkaline phosphatase (ALP) activities, compared with incubated DMCs without MMPs (experimental control) and α-MEM containing 1% FBS (negative control) (p < 0.05) (Fig. 5). One microgram per millilitre of DMCs treated with MMP-2, -8, -9, or -13 did not promote ALP activity in pulp cell cultures by day 7, compared with controls. On day 14, DMCs treated with MMP-9 and -13 also stimulated pulp cell ALP activity, similar to MMP-1 and -20 (p < 0.05) (Fig. 5). Significant differences were not observed in 1 µg/ml of DMCs treated with MMP-2 or MMP-8, or at lower concentrations (0.01–0.1 µg/ml) of MMP-treated DMCs (Supplementary Figs 7 and 8). Each MMP molecule incubated alone (0.01–1 µg/ml) was used as an additional control and showed no effect in the ALP activity assay.

### Mineralization assay

**Figure 6:**
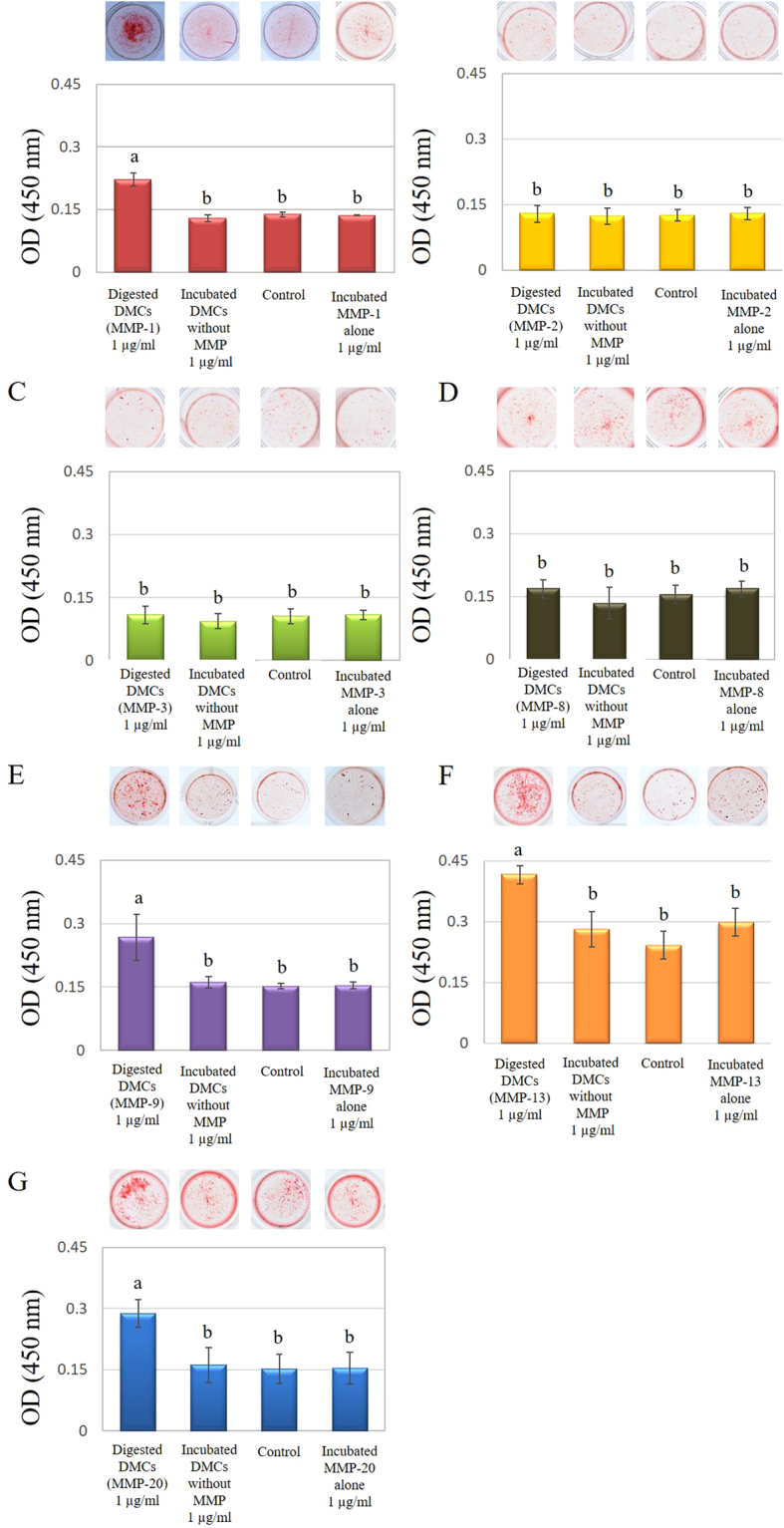
Effects of 1 µg/ml of dentin matrix components (DMCs) treated with matrix metalloproteinase (MMP)-1 (**A**), MMP-2 (**B**), MMP-3 (**C**), MMP-8 (**D**), MMP-9 (**E**), MMP-13 (**E**), or MMP-20 (**F**) on mineralized nodule formation in rat primary pulp cells. Cells were exposed to 1 µg/ml of MMP-treated DMCs, incubated DMCs without MMPs or medium alone containing 50 μg/ml of ascorbic acid and 10 mm of β-glycerophosphate for 21 days. DMCs (1 µg/ml) treated with MMP-1 (**A**), -9 (**E**), -13 (**F**), or -20 (**G**) significantly promoted mineralized nodule formation in pulp cell cultures after 21 days incubation (p < 0.05). Significant differences were not observed with DMCs treated with other MMPs, MMPs incubated alone or at lower concentrations (0.01–0.1 µg/ml) of the above-mentioned MMPs (Supplementary Figure 8). Groups with similar lower-case letters (i.e., a and b) are not significantly different. Data represent five independent experiments.

DMCs (1 µg/ml) treated with MMP-1, -9, -13, or -20 significantly promoted mineralized nodule formation in pulp cell cultures after incubation for 21 days, compared with DMCs incubated without MMPs (experimental control) and α-MEM containing 1% FBS (negative control) (p < 0.05) (Fig. 6). However, significant differences were not observed in pulp cell cultures treated with 1 µg/ml of DMCs treated by MMP-2, -3, or -8, or at lower concentrations (0.01–0.1 µg/ml) of MMP-treated DMCs (Supplementary Fig. 9). Each MMP molecule incubated alone (0.01–1 µg/ml) was used as an additional control and showed no effect in the mineralization assay.

### Evaluation of tertiary dentin formation by direct pulp capping *in vivo*

**Figure 7:**
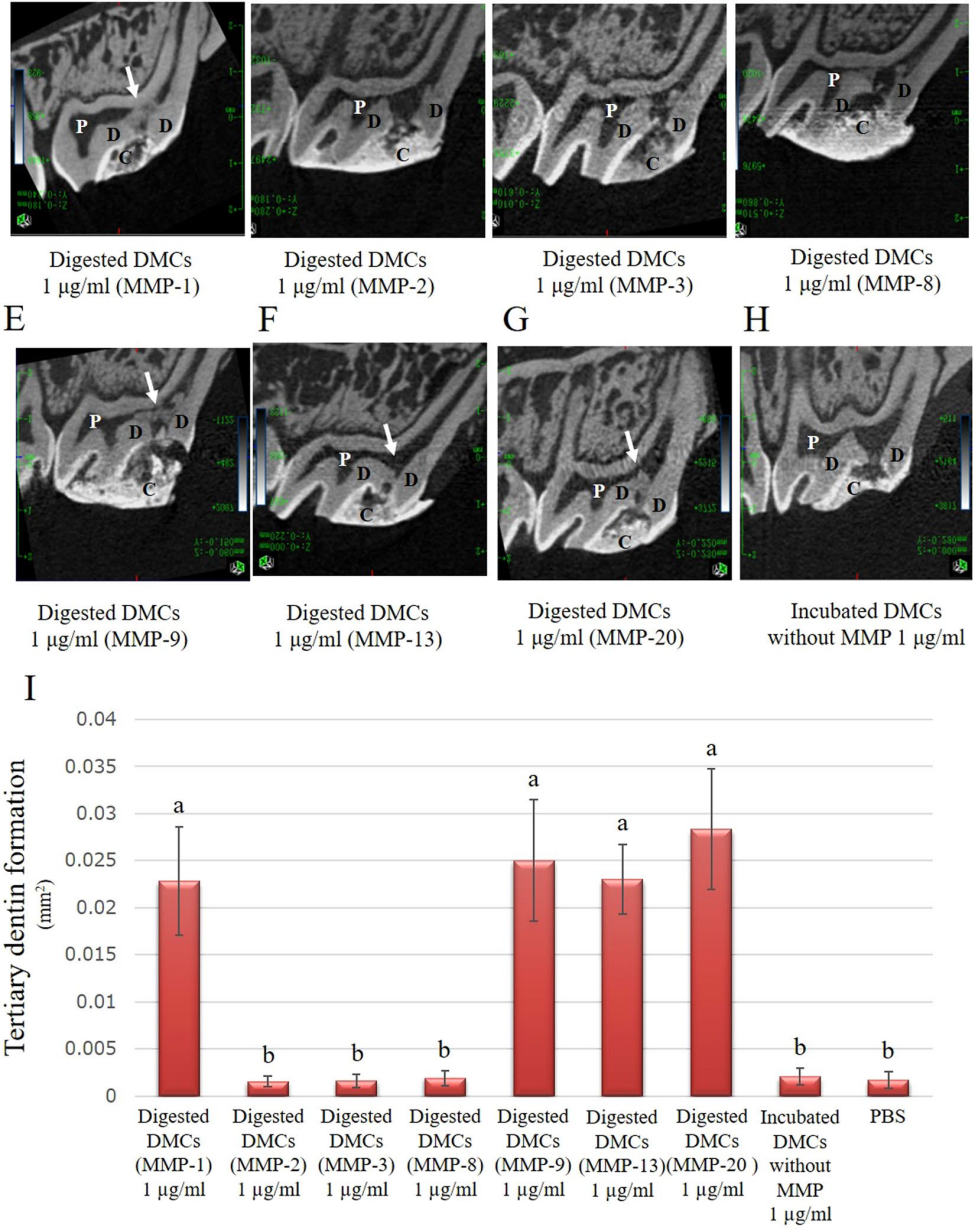
Micro-computed tomography (CT) images of tertiary dentin formation 28 days after direct pulp capping using 1 µg/ml of dentin matrix components (DMCs) treated with matrix metalloproteinase (MMP)-1 (**A**), MMP-2 (**B**), MMP-3 (**C**), MMP-8 (**D**), MMP-9 (**E**), MMP-13 (**F**), MMP-20 (**G**), or incubated DMCs without MMP (**H**). The tertiary dentin area 28 days after direct pulp capping using 1 µg/ml of DMCs digested with each MMPs showed in Fig. 7I. Quantification of tertiary dentin formation induced 1 µg/ml of DMCs treated with MMP-1, -9, -13, or -20 facilitated hard tissue formation, compared with 1 µg/ml of DMCs treated with other MMPs or incubated DMCs without MMP. One microgram per millilitre of DMCs treated with MMP-20 induced a significantly thicker tertiary dentin beneath pulp capping materials. One microgram per millilitre of DMCs treated with MMP-1, -9, and -13 induced tertiary dentin with some defect or void space beneath pulp capping materials. These data represent six independent experiments. C = cavity, D = dentin, P = pulp. White arrow = dentin bridge.

To evaluate whether DMCs treated with MMPs were able to stimulate tertiary dentin formation, a direct pulp capping experiment, which is a standard method in the dental field, was performed^5,6^. Representative micro-CT images of tertiary dentin 28 days after pulp capping by using 1 µg/ml of DMCs treated with MMP-1 (A), MMP-2 (B), MMP-3 (C), MMP-8 (D), MMP-9 (E), MMP-13 (F), MMP-20 (G), or DMCs without MMP (H) are shown in Fig. 7A–H. Quantification of tertiary dentin is shown in Fig. 7I. Tertiary dentin formation induced by 1 µg/ml of DMCs treated with MMP-1, -9, -13, or -20 was enhanced, compared with DMCs treated with other MMPs, or without MMP treatment (p < 0.05). MMP-20-treated DMCs induced hard tissue formation with fewer defects, compared with tertiary dentin induced by MMP-1, -9, or -13, which showed larger defects or a void space beneath the pulp-capping material. As a reference, representative micro-CT and histological images of tertiary dentin 28 days after pulp capping with MTA and incubation with MMP-1, MMP-2, MMP-3, MMP-8, MMP-9, MMP-13, or MMP-20 alone are shown in Supplementary Fig. 11; MTA induced highly dense tertiary dentin without significant defects.

**Figure 8:**
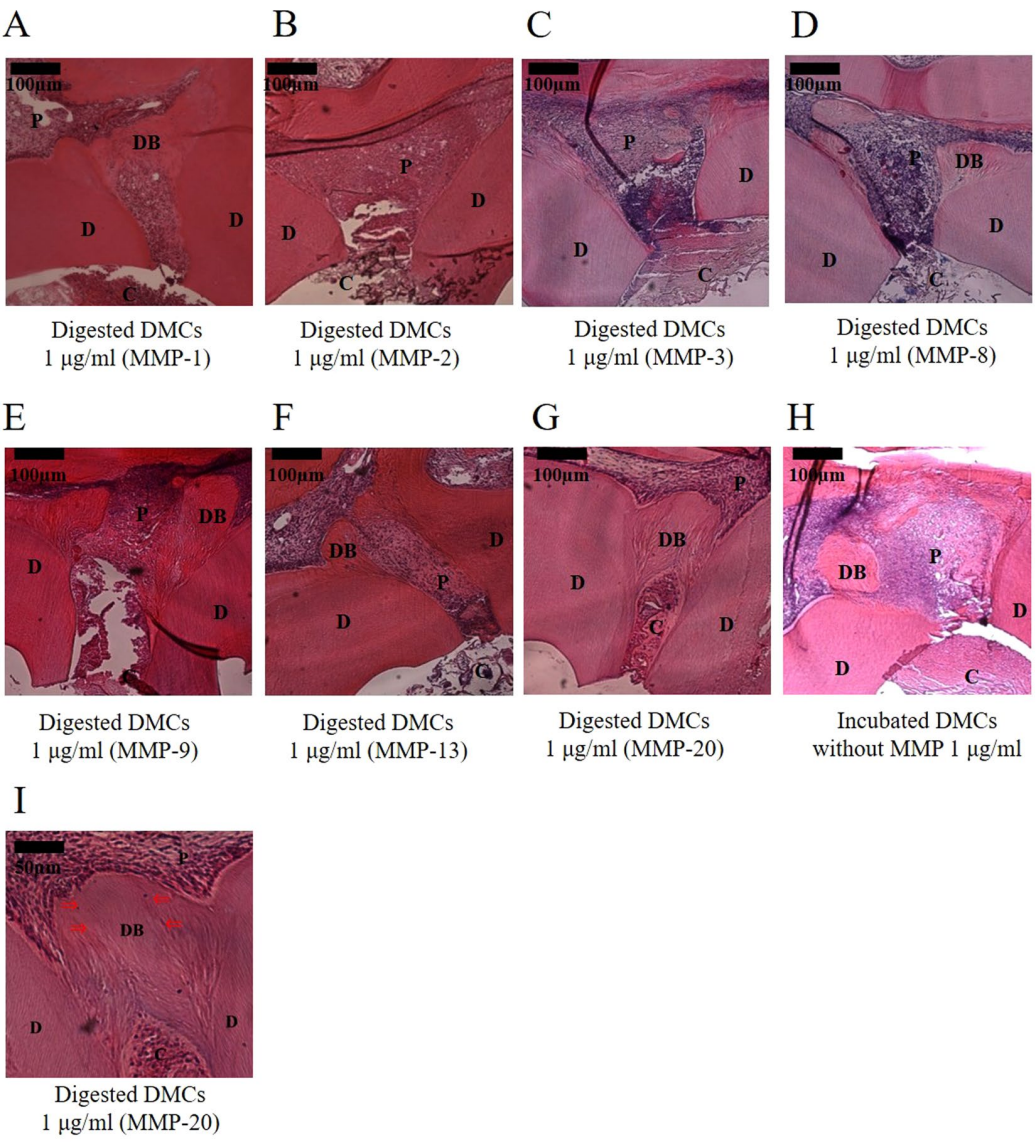
Histological images (100×) of tertiary dentin formation 28 days after direct pulp capping using 1 µg/ml of dentin matrix components (DMCs) treated with matrix metalloproteinase (MMP)-1 (**A**), MMP-2 (**B**), MMP-3 (**C**), MMP-8 (**D**), MMP-9 (**E**), MMP-13 (**F**), MMP-20 (**G**), or incubated DMCs without MMP (**H**). Higher magnification image (200×) of 1 µg/ml of DMCs digested with MMP-20 (**I**). 1 µg/ml of DMCs treated with MMP-1, -9, -13, or -20 facilitated hard tissue repair, compared with 1 µg/ml of DMCs treated with other MMPs or incubated DMCs without MMP, based on the rate of coverage of the exposed pulp by tertiary dentin (p < 0.05) (see online Supplementary Tables 1 and 2). In particular, 1 µg/ml of DMCs treated with MMP-20 induced a significantly higher condense and more regular tubular structure. Significant differences were not observed with lower concentrations (0.01–0.1 µg/ml) of the above-described MMPs. These data represent six independent experiments. Arrows indicate dentinal tubules. C = cavity, D = dentin, DB = Tertiary dentin bridge, P = pulp.

These data represent six independent experiments. Representative histological images of haematoxylin and eosin (HE)-stained teeth, 28 days after pulp capping, highlighted the positive effects of treatment with 1 µg/ml of MMP-treated DMCs (Fig. 8). One microgram per millilitre of DMCs treated with MMP-1 (Fig. 8A), MMP-9 (Fig. 8E), MMP-13 (Fig. 8F), or MMP-20 (Fig. 8G) stimulated pulp tissue repair, compared with 1 µg/ml DMCs treated with other MMPs, based on the coverage of the exposed pulp by a tertiary dentin bridge (p < 0.05) (Tables 1 and 2). Tubular structure and defects or continuous nature of tertiary dentin are commonly used to assess quality of tertiary dentin. According to these criteria, the tubular regularity of tertiary dentin formation stimulated by 1 µg/ml of DMCs treated with MMP-1, -9, -13, and -20 was superior to the formation stimulated by MMP-2, -3, or -8, as well as 1 µg/ml of DMCs without addition of MMPs (experimental control) or PBS (negative control) (p < 0.05). In particular, 1 µg/ml of DMCs treated with MMP-20 resulted in increased tertiary dentin formation and enhanced tubular structure (Fig. 8I), compared with DMCs treated with other MMPs (p < 0.05). Representative histological images of HE-stained teeth, 28 days after pulp capping with MTA are shown (Supplemental Fig. 10). Each MMP molecule incubated alone (0.01–1 µg/ml) was used as an additional control and showed no effect on the direct pulp capping (Supplemental Fig. 10).

**Table 1:** Criteria used for histological analysis of tertiary dentinogenesis.

Histological evaluation	
Grade	Dentin bridge formation
0	No dentin bridge formation
1	Slight dentin bridge formation (dentin bridge formation covers 1/3 of exposed pulp)
2	Incomplete dentin bridge formation (dentin bridge formation covers 2/3 of exposed pulp)
3	Complete dentin bridge formation (dentin bridge formation completely covers exposed pulp)

**Table 2:** Grading results of the dentin bridge formation following the criteria of pulp histological consecutive sections 4 weeks after direct pulp capping (n = 6 per group).

Grade	Dentin bridge formation			
Material	0	1	2	3
Digested DMCs (MMP-1)^*^	1	2	2	1
Digested DMCs (MMP-2)	5	1	0	0
Digested DMCs (MMP-3)	5	1	0	0
Digested DMCs (MMP-8)	5	1	0	0
Digested DMCs (MMP-9)^*^	2	2	1	1
Digested DMCs (MMP-13)^*^	2	1	2	1
Digested DMCs (MMP-20)^*,**^	0	0	4	2
Incubated DMCs without MMP	3	2	1	0
PBS	6	0	0	0

^*^1 µg/ml of DMCs treated with MMP-1, MMP-9, MMP-13, or MMP-20 stimulated pulp tissue repair, compared with 1 µg/ml DMCs treated with other MMPs. ^**^In particular, 1 µg/ml of DMCs treated with MMP-20 resulted in significantly increased tertiary dentin formation, compared with all other MMPs. DMCs: dentin matrix components, PBS: phosphate-buffered saline; MMP, matrix metalloproteinase.

## Discussion

Traditional direct pulp capping materials, such as Ca (OH)_2_ or MTA, have a 60%–80% success rate, as reported in clinical studies^4,23,24^. However, these materials were developed on a more empirical basis, rather than through specific understanding of the wound-healing mechanisms of the dentin-pulp complex. Recent studies have aimed to introduce bio-mimicked tissue healing with growth factors as a direct pulp-capping material^25–27^. However, limited effects have been observed regarding the healing of wounded pulp tissues as the wound healing mechanisms of the dentin-pulp complex remain not entirely clear.

MMPs are calcium- and zinc-dependent, host-derived enzymes^28^ that can hydrolyse ECM components^29^. ECM integrity is affected by MMPs and TIMPs, which play critical roles during matrix remodelling^9–11^. Several MMPs have been reported to be sequestered in mineralized dentin^30^, and recent studies revealed that host-derived MMPs contribute to the breakdown of collagenous matrices during dental caries^30^. Conversely, MMPs have been reported to be involved in wound healing processes, such as cell growth, cell migration, tissue remodelling and angiogenesis^31,32^. We also previously reported upregulated MMP gene expression after pulp injury^8^ and MMPs have been detected in the dentin matrix in an inactive state^33,34^. The distribution of MMP molecules has been reported at deeper sites within the dentin, where they exhibit gradients of activity^35–37^. The presence of these molecules within the dentin-pulp complex enable them to activate DMCs released by bacterial acids or restorative materials. These activated molecules may subsequently act in biological defence and repair processes within the tooth. Therefore, in the present study, we focused on MMPs that might facilitate matrix remodelling of dental pulp tissues or digested DMCs which promote wound healing^21,37^.

Dentin is a major component of the tooth and covers the dental pulp. It is considered to be part of the ECM of the combined dentin-pulp complex, rather than being a distinct tissue, as it originates from the same mesenchymal tissue^38,39^. However, the complete range of functions of dentin is still under investigation. During caries progression, dentin is demineralized by acids secreted by caries-associated bacteria, and DMCs may be digested enzymatically during this process^40^. Therefore, we investigated the effects of digested DMCs on wound healing of the dentin-pulp complex both *in vitro* and *in vivo*.

Protein analysis by SDS-PAGE showed DMCs were digested with MMPs-1, -2, -3, -8, -9, -13, and -20. The resultant protein profiles differed among MMPs (Fig. 1), which was as expected because of their differing substrate specificities. Generally, MMP-1 has been considered as a collagenase and MMP-2 is reported as a gelatinase. However, MMP-1 is also able to degrade proteoglycans^41^ and MMP-2 reportedly can degrade and activate the DMP-1 protein, which is a key dentin matrix signalling protein^42^. In addition, non-canonical substrates have been identified as targets of these MMPs^41^. Similarly, MMP20 has been recognized as enamelysin and cleaves enamel-related proteins; however, further substrates could exist. Recently, MMP20 was reported to associate with the activation of DSPP, which is a member of the small integrin-binding ligand, N-linked glycoprotein (SIBLING) family^43^. Several previous reports have demonstrated that various proteins from the SIBLING family, along with several growth factors, are present in DMCs^44–47^. Therefore, DMCs may contain SIBLING substrates for MMPs.

Subsequently, we performed SDS-PAGE analysis and confirmed that DMCs extracted with EDTA could react and respond to the MMP molecules. These results suggested that MMPs may hydrolyse or facilitate dentin decomposition, consistent with the findings from these previous studies^48^.

Next, we examined the effects of DMCs digested with MMPs on the functions of primary pulp cells *in vitro*. DMCs digested with some MMPs promoted angiogenesis, migration, proliferation, migration, osteogenic differentiation and hard tissue formation. However, MMP molecules that promote wound healing in dental pulp cells may operate differently *in vivo* and the Cellular responses may be due to the target cells expressing different substrate binding sites for the MMP-digested DMCs. These *in vivo* outcomes may indicate variations in functionality of the digested products from the same substrates according to the enzymes used to digest them; and this variation also agrees with previous reports^49^. Indeed, whilst we observed that DMCs digested with MMPs up-regulated some pulp cell responses *in vitro* (as described above) which associate with the wound healing response in the pulp tissue, it was however important to directly investigate whether MMP-digested DMCs invoked tissue healing responses *in vivo*, by using a direct pulp capping approach. Such an approach enables the evaluation of a more complete and simulated form of dental tissue repair and may also identify translational opportunities. Thus, we investigated the effects of digested DMCs on wound healing in pulp tissue by using direct pulp capping *in vivo*. The result of micro-CT analysis of hard tissue formation indicated that DMCs digested with MMP-20 induced highly condensed tertiary dentin beneath the pulp-capping materials applied (Fig. 7H,I) and histological images from the same specimen showed appropriate tubular dentin morphology (Fig. 8I). In contrast, DMCs digested with other MMPs induced tertiary dentin with more defects and void structures (Fig. 7A,E,F; Tables 1 and 2). These results suggest that MMP20 may play an essential role in the pulpal healing process, compared with other MMPs *in vivo*.

Given that wound healing *in vivo* is affected by a variety of complex and interacting factors, the results of *in vitro* experiments might not always mirror the *in vivo* processes. Furthermore, the source of MMPs *in vivo* is unclear and could be derived from variety of sources and locations, such as resident cells within the pulp and/or the inflammatory infiltrate. A further potential explanation for the differences observed *in vivo* and *in vitro* may be due to the presence of other environmental factors, as it has been shown that ECM digested with several enzymes stimulated different actions, according to the environment studied^50^.

Most reports concerning MMP-20 have found that this enzyme appears to be predominantly expressed within the tooth^51,52^. Indeed, during tooth development, MMP-20 may contribute to enamel formation by digesting enamel-related proteins^53^. Additionally, MMP-20 is present in the dentin matrix and is expressed by odontoblasts in mature teeth and activated by dental caries progression^17^. Others have reported that MMP-20 is present in carious dentin compared with sound dentin^33^. More specifically these reports relate to the localization of MMP20 in the dentin-pulp complex; however, there appear to be no reports of its biological role in this location. Data from the present study indicate that MMP-20 might be involved in a specific pulpal repair system enabling tertiary dentin formation.

For *in vitro* and *in vivo* experiments, MMP molecules alone were also used to investigate their direct effects; however, no direct effects on pulp cell function or pulpal tissue repair were observed. This outcome is likely due to the MMP molecule exerting no direct effect on these responses, or the inability to generate sufficient substrates in this environment due to the target molecules being embedded within the mineralized ECM. In addition, the most effective ratio of MMPs and DMCs is unknown. The concentration of MMPs in this study may not reflect the true biological environment as is present in dental caries. However, this study does indicate a potential role for MMP molecules in modifying or improving the local healing response of the dentin-pulp complex, via its action on DMCs. This model may therefore provide novel insight enabling the future elucidation of the mechanism involved in pulpal healing.

The different MMPs may also function at different doses; therefore, future studies are needed to determine optimal doses required. As MMPs have been reported to directly upregulate dental pulp through protease-activated receptors^54,55^, further investigation is also necessary to address this. In addition, other enzymes (e.g., serine or cysteine proteinases) are present in the dentin matrix^56^ and these proteinases may further degrade DMCs^57^. Thus, the effects of other endogenous enzymes during repair of the dentin-pulp complex should be examined.

**Figure 9:**
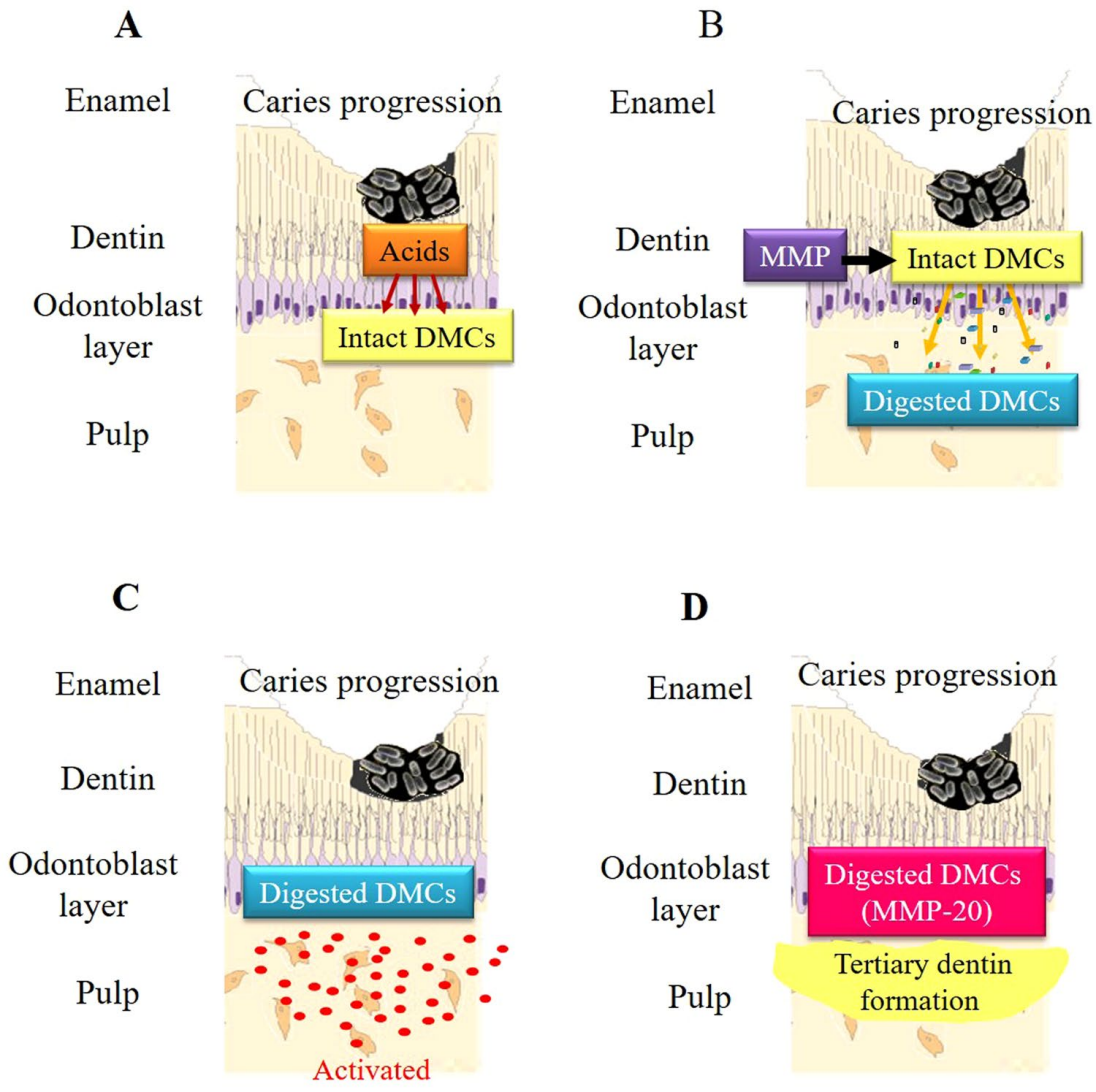
Proposed model for wound healing in pulp tissue following dental caries. Dentin decalcification initially releases matrix metalloproteinases (MMPs) from the dentin matrix during caries progression (**A**). The dentin matrix is then digested with MMPs (**B**). Dental pulp cells are subsequently activated by digested dentin matrix components (DMCs) (**C**). DMCs digested by MMPs, particularly MMP-20, stimulate wound healing in the dentin-pulp complex (**D**).

Based on our findings, host-derived MMP may be involved in the wound healing process of the dentin-pulp complex (Fig. 9). During caries progression, initially enamel and then dentin is decalcified by bacterial acids and the organic dentin matrix is exposed (Fig. 9A). Subsequently, the dentin matrix is digested with MMPs, which may be present endogenously (Fig. 9B), and dental pulp cells are activated by these MMP-digested DMCs (Fig. 9C). DMCs digested with MMP-1, -9, -13, and -20 facilitated wound healing of the dentin-pulp complex in our system (Fig. 9D). To our knowledge, this study is the first to examine the wound healing potential of the dentin-pulp complex by using DMCs digested with MMP-1, -2, -3, -8, -9, -13, and -20; the data identify a significant role for MMP-20 in mature teeth. Further studies are necessary to analyse the components generated by digested DMCs and identify which molecules are critical for wound healing of this tissue.

## Methods

### Preparation of DMCs digested with MMPs

Human dentin specimens were prepared from extracted sound non-carious teeth (erupted, permanent dentition) collected from patients of both genders and all ages (generally 16–40 years) attending the Oral Surgery Department at Birmingham Dental Hospital (Birmingham, UK). Informed consent was obtained from all subjects. The protocols and all experimental procedures were approved by and performed in accordance with the relevant guidelines and regulations of the ethics approval of the University of Birmingham School of Dentistry Tooth Bank (Ethics approval no. 90/H0405/33) as described in a previous report^58^.

Briefly, after the tooth was extracted, pulp tissue and enamel were removed from the tooth by using dental instruments. The remaining dentin was pulverized into a powder by using a percussion mill (Spex 6700 Freezer/Mill, Glen Creston Ltd, Stanmore, UK), then cooled with liquid nitrogen and filtered through a 60-μm mesh sieve. Powdered dentin was treated with a 10% (w/v) EDTA solution containing proteinase inhibitors, 10 mM N-ethylmaleimide (Sigma-Aldrich, Dorset, UK), and 5 mM phenylmethylsulfonyl fluoride (Sigma-Aldrich) for 14 days with agitation at 4 °C. The extraction solution was changed daily after centrifuging at 3,000 rcf for 10 min and the supernatant was collected. The pooled extraction supernatants were dialysed extensively against distilled water for 7 days and lyophilized by using a freeze dryer (Modulyo, London, UK). A dialysis membrane with a molecular weight cut-off of approximately 12,000 kDa was used to maintain consistency among dentin matrix preparations^59,60^. The lyophilized powder was collected as intact DMCs.

Activation of MMPs was performed by treated with p-aminophenylmercuric acetate (APMA; Sigma-Aldrich) in the following experiments, according to manufacturer’s protocol. DMCs were treated with MMPs as follows: 20 µl of each MMP (50 µg/ml) (MMP-1, -2, -3, -8, -9, -13, and -20) were added to 500 µl of intact DMCs (10 μg/ml) and incubated for 1 h or 24 h at 37 °C in PBS (pH 7.4, Sigma-Aldrich, Dorset, UK). Ten micrograms per millilitre of DMCs was resuspended to 0.01–1 µg/ml of DMCs using each media following assay. MMP-1, -2, -3, -8, -9, and -13 were purchased from R&D Systems, Inc. (Minneapolis, MN, USA) and MMP-20 was purchased from Funakoshi Co. Ltd. (Tokyo, Japan). A 0.22-µm porous filter (Millex^®^ GP, Merck, Billerica, MD, USA) was used for sterilization of the DMCs.

### SDS-PAGE analysis

To evaluate the protein profiles of MMP-treated DMCs or incubated DMCs without MMP addition, SDS-PAGE analysis was performed. After dissolving 1 μg/ml of digested DMCs in phosphate-buffered saline (PBS; Sigma-Aldrich), incubated DMCs without MMP in PBS, or an MMP-only solution of a specific MMP dissolved in PBS in loading buffer (Life Technologies, Carlsbad, CA, USA), the solution was denatured at 70 °C for 10 min. Then, 2 μl of the solution were loaded onto 4%–12% Bis-Tris gels (NuPAGE, Life Technologies) and electrophoresed at 200 V, 45 mA for 40 min with SDS running buffer (Life Technologies). The gels were stained by using a silver staining kit (Silver Xpress^®^, Life Technologies) according to the manufacturer’s instructions.

### Cell culture

All experimental procedures were performed in accordance with the relevant guidelines and regulations of Osaka University, and all experimental procedures were conducted with approval by the Ethical Guidelines Committee for Animal Care of Osaka University Graduate School of Dentistry (No. 23–005–1). Primary rat pulp cells were collected from 6-week-old male Wistar rats (CLEA Japan, Inc., Tokyo, Japan), as has been previously described^61^. After upper and lower incisors were extracted under intraperitoneal anaesthesia with sodium pentobarbital solution (Somnopentyl, Kyoritsu Seiyaku Corp., Tokyo, Japan) (30 mg/kg), pulp tissue was collected and minced by using a #12 scalpel and treated with 0.025% trypsin/0.01% EDTA solution (Sigma-Aldrich) for 30 min. Dissociated pulp cells were then cultured in α-MEM (Life Technologies) supplemented with 20% (v/v) fetal bovine serum (FBS; Sigma-Aldrich) and 10 μg/ml of penicillin-streptomycin (Sigma-Aldrich) at 37 °C in a humidified incubator with 5% CO_2_ for 10 days. Primary pulp cell cultures of passage 1 were used in subsequent experiments.

### Angiogenesis assay

To evaluate the angiogenic effects of DMCs treated with MMPs, an endothelial tube formation assay was performed with the Angiogenesis Kit (Kurabo, Osaka, Japan). Human umbilical vein endothelial cells were co-cultured with human fibroblasts as a feeder layer containing 0.01–1 µg/ml of MMP-treated DMCs or incubated DMCs without MMP as an experimental control in angiogenesis induction medium (Kurabo). After incubation for 11 days, cells were fixed with 70% (v/v) ethanol for 30 min, then immunostaining with CD31 (mouse anti-human CD31, Kurabo) to visualize endothelial cells of blood vessels. The acquired images were analysed by using Angiogenesis Image Analyzer V.2.0.5 (Kurabo) software to quantify the newly formed tubular formation, including vessel number, branch length and branch point in blood vessel-like structures (n = 3). Angiogenesis induction medium containing 20 μM/L of suramin was used as a negative control and recombinant human VEGF (2 ng/ml) was used as a positive control. The exact mechanism of suramin as an inhibitor of angiogenesis is not known; however, its non-specific interactions with numerous growth factors and angiogenic factors, such as basic fibroblast growth factor (b-FGF), platelet-derived growth factor, transforming growth factor, and VEGF, have been shown^62^. VEGF, as a promoter of angiogenesis, has been reported to regulate intracellular functions and activities by binding to receptors^63^. Each MMP molecule incubated alone (0.01–1 µg/ml) was used as an additional control.

### Migration assessment by wound scratch assay

An *in vitro* wound scratch healing model was used to investigate cell migration. Rat primary pulp cells (5 × 10^4^ cells) were seeded onto 6-well cell culture dishes (Becton Dickinson and Company, Franklin Lake, NJ, USA) containing α-MEM supplemented with 10% FBS. When cells became sub-confluent, the medium was changed to α-MEM supplemented with 1% (v/v) FBS and cells were cultured for an additional 24 h. Then, 10 µg/ml of mitomycin-C (Calbiochem, San Diego, CA, USA) was applied to the medium for 30 min to remove the effect of proliferation and determine more specifically the role of migration in closure of the scratch wound by the cells. The experimental approach used here is based on previous studies, which have explored the roles of proliferation and migration in the wound closure assays^64^. An artificial scratch wound was created by disrupting the monolayer using a sterile plastic pipette tip. Medium containing 0.01–1 µg/ml of MMP-treated DMCs or incubated DMCs without MMP as an experimental control were then added and cells were cultured for 36 h to allow migration into the wounded area. Cells not treated with DMCs served as a negative control. Each MMP molecule incubated alone (0.01–1 µg/ml) was used as an additional control. Eight points were randomly selected to assess migration; the distances of the cells from the edge of the scratch wound were averaged and statistically evaluated by microscopic observation (Fig. 3A) and compared with controls by using ImageJ software (Version 1.47, National Institutes of Health, Bethesda, MD, USA).

### Migration assessment by trans-well assay

Cell migration (chemotaxis) was also evaluated by using a Boyden chamber (membrane pore size = 8 µm) (Cytoselect^®^, Cell Biolabs, San Diego, CA, USA)^65^. Rat primary pulp cells were seeded into the upper chamber in α-MEM containing 1% (v/v) FBS at a density of 2.0 × 10^4^ cells, and MMP-treated DMCs or incubated DMCs without MMP (0.01–1.0 µg/ml) as an experimental control in α-MEM containing 1% FBS were added to the lower chamber. After 2 h incubation, cells that passed through the membrane from the upper to lower chamber were removed by using cell detachment buffer (Cell Biolabs) for 30 min. Then, CyQuant^®^ GR dye (Cell Biolabs) was added to the lower chamber for fluorescent staining of migratory cells. Migration was quantified by using a microplate reader (ARVO MX, PerkinElmer, Waltham, MA, USA) at 485 nm/535 nm fluorescence intensity (n = 8 for each group). Cells not treated with DMCs in the lower chamber served as a negative control. Each MMP molecule incubated alone (0.01–1 µg/ml) was used as an additional control.

### Cell proliferation assay

To evaluate the effects of MMP-treated DMCs on the growth of rat primary pulp cells, the WST-1 assay was performed. Rat primary pulp cells (1.0 × 10^4^ cells) were cultured in α-MEM supplemented with digested DMCs (0.01–1.0 µg/ml) or incubated DMCs without MMP (0.01–1.0 µg/ml) as an experimental control for 5 days. Then, WST-1 reagent (Roche, Basel, Switzerland) was added and cells were incubated for 2 h. The fluorescence at 450 nm was measured using a microplate reader (ARVO MX) to evaluate cell proliferation (n = 6 for each group). Cells not treated with DMCs served as negative controls. Each MMP molecule incubated alone (0.01–1 µg/ml) was used as additional control.

### ALP activity

Alkaline phosphatase (ALP) activity was measured to investigate the effects of digested DMCs on pulp cell differentiation^66^. Rat primary pulp cells were cultured with 0.01–1 µg/ml MMP-treated DMCs or incubated DMCs without MMP as an experimental control in α-MEM supplemented with 10 mM β-glycerophosphate (Sigma-Aldrich), 50 µg/ml ascorbic acid (Sigma-Aldrich), and 10% (v/v) FBS (differentiation induction medium) at a density of 2.0 × 10^4^ cells/well. After 7- or 14-day incubation, ALP activity was measured with the Alkaline Phosphate Substrate Kit (Bio-Rad Laboratories, Hercules, CA, USA) according to the manufacturer’s instructions. Cells not treated with DMCs served as negative controls (n = 6 for each group). Each MMP molecule incubated alone (0.01–1 µg/ml) was used as an additional control.

### Mineralization assay

Mineralization of rat primary pulp cells was evaluated by alizarin red staining (PG Research, Tokyo, Japan). Rat primary pulp cells (5.0 × 10^4^ cells) were incubated in differentiation induction medium with 0.01–1 µg/ml of MMP-treated DMCs or incubated DMCs without MMPs as an experimental control for 21 days. Then, cells were fixed in 10% neutral buffered formalin and mineralized nodules were stained with alizarin red. The bound dye was quantified with a mineralization assay kit (PG Research) by measuring absorbance at 405 nm by using a microplate reader (ARVO MX). Specimens cultured in mineralization medium that were not treated with DMCs served as negative controls (n = 6 for each group). Each MMP molecule incubated alone (0.01–1 µg/ml) was used as an additional control.

### Direct pulp capping using rat teeth *in vivo*

The effects of MMP-treated DMCs on wound healing of the dentin-pulp complex were examined by direct pulp capping in 8-week-old male Wistar rats (CLEA Japan). After general anaesthesia, the pulp was exposed on the occlusal surfaces of maxillary first molars by using a #1 round carbide bur (Dentsply Maillefer, Ballaigues, Switzerland), as previously described^8,62^. The size of the cavity measured approximately 1 mm in depth and 0.4 mm in diameter. The exposed pulp tissue was rinsed with saline and dried, then directly covered with a gelatine sponge (Spongel^®^, Astellas Pharma Inc, Tokyo, Japan) containing 20 µl of MMP-treated DMCs (0.01–1 µg/ml), or incubated DMCs without MMP as an experimental control. ProRoot MTA (Dentsply, Tulsa, OK, USA) was used as a positive control^3,6,66^. Each MMP molecule incubated alone (0.01–1 µg/ml) was used as an additional control. The cavity was then sealed with glass ionomer cement (Fuji IX, GC, Tokyo, Japan). Specimens with a gelatine sponge containing 20 µl of PBS served as negative controls (n = 6 for each group).

Twenty-eight days after pulp capping, rats were perfused with physiological saline followed by paraformaldehyde solution (Nacalai Tesque, Kyoto, Japan). Pulp-capped teeth were removed en bloc with surrounding maxillary bone and immersed in the same fixative for an additional 24 h. The induced tertiary dentin (n = 6 in each group) was analysed by using a micro-CT scanner (R_mCT2, Rigaku, Tokyo, Japan) at settings of 90 kV and 160 µA with a scanning resolution of 20 μm intervals in individual image. After scanning, the area of induced tertiary dentin of each specimen was quantitatively analysed by using three-dimensional reconstruction images for bone (TRI/3D-BON; Ratoc System Engineering, Tokyo, Japan) with reference to previous report^67,68^. After confirming the consistency between micro-CT and histological images, image analysis was performed. Teeth were demineralized in a 10% (v/v) citric- 22.5% (v/v) formic acid solution (Wako Pure Chemicals, Osaka, Japan) for 1 week at 4 °C. The specimens were prepared by the previous protocol of graded alcohol dehydration, followed by paraffin embedding, and sagittal sections were prepared at a thickness of 5 µm for HE staining^69^. Wound healing of the pulp tissue was quantified based on tertiary dentin formation, as previously described^70^. Namely, conventional histopathological methods were used to analyse six consecutive sections from each sample. Defects in tertiary dentin could be generally observed around the centre of exposed pulp area due to tertiary dentin formation that began at the periphery of the exposed pulp tissue. Subsequently, we selected and evaluated six consecutive images from the central area of the tertiary dentin to determine the quality of tertiary dentin with reference to micro-CT images, based on previously published histological criteria. The criteria for histological evaluation were derived from previous publications^71–73^ and are shown in Table 1. For the histological evaluation, two independent observers were trained to evaluate histological features by using the designated criteria with 95% consistency referring to micro-CT images. If there was disagreement between the two observers, consensus was reached by discussion.

### Statistical analysis

Significant differences were evaluated by one-way ANOVA with Tukey’s post-hoc test, the Kruskal-Wallis test, or the Steel-Dwass test. P values < 0.05 were considered statistically significant.

### Data availability

The datasets generated during and/or analysed during the current study are available from the corresponding author on request.

## Electronic supplementary material

Supplementary Figure

## References

[1] George TH (2011). Dental pulp and dentin tissue engineering and regeneration: advancement and challenge. Front Biosci..

[2] He W (2014). Lipopolysaccharide enhances Wnt5a expression through toll-like receptor 4, myeloid differentiating factor 88, phosphatidylinositol 3-OH kinase/AKT and nuclear factor kappa B pathways in human dental pulp stem cells. J Endod..

[3] Hilton TJ, Ferracane JL, Mancl L (2013). For Northwest Practice-based Research Collaborative in Evidence-based Dentistry (NWP), Comparison of Ca(OH)2 with MTA for direct pulp capping: a PBRN randomized clinical trial. J Dent Res..

[4] Mente J (2014). Treatment outcome of mineral trioxide aggregate or calcium hydroxide direct pulp capping: long-term results. J Endod..

[5] Ishimoto K (2015). Topical application of lithium chloride on the pulp induces dentin regeneration. PLoS One..

[6] Zhang J (2015). Promotion of Dental Pulp Cell Migration and Pulp Repair by a Bioceramic Putty Involving FGFR-mediated Signaling Pathways. J Dent Res..

[7] Pratia C, Gandolfi MG (2015). Calcium silicate bioactive cements: Biological perspectives and clinical applications. Dent Mater..

[8] Yoshioka S (2013). Activation of the Wnt/β-catenin pathway and tissue inhibitor of metalloprotease 1 during tertiary dentinogenesis. J Biochem..

[9] Kessenbrock K, Wang CY, Werb Z (2015). Matrix metalloproteinases in stem cell regulation and cancer. Matrix Biol..

[10] Duarte S, Baber J, Fujii T, Coito AJ (2015). Matrix metalloproteinases in liver injury, repair and fibrosis. Matrix Biol..

[11] Fields GB (2015). New strategies for targeting matrix metalloproteinases. Matrix Biol..

[12] Deryugina EI, Quigley JP (2015). Tumor angiogenesis: MMP-mediated induction of intravasation and metastasis sustaining neovasculature. Matrix Biol..

[13] Arpinoa V, Brocka M, Gill SE (2015). The role of TIMPs in regulation of extracellular matrix proteolysis. Matrix Biol.

[14] Hattori N (2009). MMP-13 plays a role in keratinocyte migration, angiogenesis, and contraction in mouse skin wound healing. Am J Pathol..

[15] Adair-Kirk TL (2003). A site on laminin alpha 5, AQARSAASKVKVSMKF, induces inflammatory cell production of matrix metalloproteinase-9 and chemotaxis. J Immunol..

[16] Brennan EP, Tang X-H, Stewart-Akers AM, Gudas LJ, Badylak SF (2008). Chemoattractant activity of degradation products of fetal and adult skin extracellular matrix for keratinocyte progenitor cells. J Tissue Eng Regen Med..

[17] Sulkala M, Larmas M, Sorsa T, Salo T, Tjäderhane L (2002). The localization of matrix metalloproteinase-20. J Dent Res..

[18] Yamakoshi Y (2006). Dentin sialophosphoprotein is processed by MMP-2 and MMP-20 *in vitro* and *in vivo*. J Biol Chem..

[19] Hannas AR, Pereira JC, Granjeiro JM, Tjäderhane L (2007). The role of matrix metalloproteinases in the oral environment. Acta Odontol Scand..

[20] Väänänena A (2004). Expression of collagen XVIII and MMP-20 in developing teeth and odontogenic tumors. Matrix Biol..

[21] Mazzoni A (2015). Role of dentin MMPs in caries progression and bond stability. J Dent Res..

[22] Mitsiadis TA, Feki A, Papaccio G, Catón J (2011). Dental pulp stem cells, niches, and notch signaling in tooth injury. Adv Dent Res..

[23] Bjørndal L (2010). Treatment of deep caries lesions in adults: randomized clinical trials comparing stepwise vs. direct complete excavation, and direct pulp capping vs. partial pulpotomy. Eur J Oral Sci..

[24] Aguilar P, Linsuwanont P (2011). Vital pulp therapy in vital permanent teeth with cariously exposed pulp: a systematic review. J Endod..

[25] Iohara K (2004). Dentin regeneration by dental pulp stem cell therapy with recombinant human bone morphogenetic protein 2. J Dent Res..

[26] Decup F (2000). Bone sialoprotein-induced reparative dentinogenesis in the pulp of rat’s molar. Clin Oral Investig..

[27] Yang IS (2010). Tertiary dentin formation after direct pulp capping with odontogenic ameloblast-associated protein in rat teeth. J Endod..

[28] Amălinei C, Căruntu ID, Bălan RA, Rom J (2007). Biology of metalloproteinases. Morphol Embryol.

[29] Brinckerhoff CE, Matrisian LM (2002). Matrix metalloproteinases: a tail of a frog that became a prince. Nat Rev Mol Cell Biol..

[30] Hedenbjörk-Lagera A, Hamberga K, Pääkkönenb V, Tjäderhaneb L, Ericson D (2016). Collagen degradation and preservation of MMP-8 activity in human dentine matrix after demineralization. Arch Oral Biol..

[31] Giannelli G, Falk-Marzillier J, Schiraldi O, Stetler-Stevenson WG, Quaranta V (1977). Induction of cell migration by matrix metalloprotease-2 cleavage of laminin-5. Science..

[32] Palosaari H (2003). Expression profile of matrix metalloproteinases (MMPs) and tissue inhibitors of MMPs in mature human odontoblasts and pulp tissue. Eur J Oral Sci..

[33] Shimada Y, Ichinose S, Sadr A, Burrow MF, Tagami J (2009). Localization of matrix metalloproteinases (MMPs-2, 8, 9 and 20) in normal and carious dentine. Aust Dent J..

[34] Hedenbjörk-Lager A (2015). Caries correlates strongly to salivary levels of matrix metalloproteinase-8. Caries Res..

[35] Niu LN (2011). Localization of MMP-2, MMP-9, TIMP-1, and TIMP-2 in human coronal dentine. J Dent..

[36] Sulkala M (2004). Matrix metalloproteinase-13 (MMP-13, collagenase-3) is highly expressed in human tooth pulp. Connect Tissue Res..

[37] Kato MT (2011). Activity of matrix metalloproteinases in bovine versus human dentine. Caries Res..

[38] Mazzoni A (2009). A. *et al*. Immunohistochemical identification of MMP-2 and MMP-9 in human dentin: correlative FEI-SEM/TEM analysis. J Biomed Mater Res A..

[39] Sulkala M (2007). Matrix metalloproteinase-8 (MMP-8) is the major collagenase in human dentin. Arch Oral Biol..

[40] Chaussain-Miller C, Fioretti F, Goldberg M, Menashi S (2006). The role of matrix metalloproteinases (MMPs) in human caries. J Dent Res..

[41] Zhang SC, Kern M (2009). The role of host-derived dentinal matrix metalloproteinases in reducing dentin bonding of resin adhesives. Int J Oral Sci..

[42] Chaussain C (2009). MMP2-cleavage of DMP1 generates a bioactive peptide promoting differentiation of dental pulp stem/progenitor cell. Eur Cell Mater..

[43] Saxena G, Koli K, de la Garza J, Ogbureke KU (2015). Matrix metalloproteinase 20-dentin sialophosphoprotein interaction in oral cancer. J Dent Res..

[44] Leaver AG, Price R, Smith AJ (1978). The insoluble fraction isolated after digestion of demineralized human dentine matrix with collagenase. Arch Oral Biol..

[45] Smith AJ, Leaver AG (1978). The effects of periodate degradation and collagenase digestion on the organic matrix of human dentine. Arch Oral Biol..

[46] Salehi S, Cooper P, Smith A, Ferracane J (2016). Dentin matrix components extracted with phosphoric acid enhance cell proliferation and mineralization. Dent Mater..

[47] Duncan HF, Smith AJ, Fleming GJ (2017). Release of bio-active dentine extracellular matrix components by histone deacetylase inhibitors (HDACi). Int Endod J..

[48] Sternlicht MD, Werb Z (2001). How matrix metalloproteinases regulate cell behavior. Annu Rev Cell Dev Biol..

[49] Suhr F, Brixius K, Bloch W (2009). Angiogenic and vascular modulation by extracellular matrix cleavage products. Curr Pharm Des..

[50] Ricard-Blum S, Salza R (2014). Matricryptins and matrikines: biologically active fragments of the extracellular matrix. Exp Dermatol..

[51] Caterina JJ (2002). Enamelysin (matrix metalloproteinase 20)-deficient mice display an amelogenesis imperfecta phenotype. J Biol Chem..

[52] Shin M (2014). Matrix metalloproteinase-20 over-expression is detrimental to enamel development: a Mus musculus model. PLoS One..

[53] Lu Y (2008). Functions of KLK4 and MMP-20 in dental enamel formation. Biol Chem..

[54] Syggelos SA, Aletras AJ, Smirlaki I, Skandalis SS (2013). Extracellular matrix degradation and tissue remodeling in periprosthetic loosening and osteolysis: focus on matrix metalloproteinases, their endogenous tissue inhibitors, and the proteasome. Biomed Res Int..

[55] Trivedi V (2009). Platelet matrix metalloprotease-1 mediates thrombogenesis by activating PAR1 at a cryptic ligand site. Cell..

[56] Tersariol IL (2010). Cysteine cathepsins in human dentin-pulp complex. J Endod..

[57] Lacruz RS (2011). Chymotrypsin C (caldecrin) is associated with enamel development. J Dent Res..

[58] Tomson PL (2007). Dissolution of bio-active dentine matrix components by mineral trioxide aggregate. J Dent..

[59] Bègue-Kirn C (1992). Effects of dentin proteins, transforming growth factor beta 1 (TGF beta 1) and bone morphogenetic protein 2 (BMP2) on the differentiation of odontoblast *in vitro*. Int J Dev Biol..

[60] Smith AJ (2012). Dentine as a bioactive extracellular matrix. Arch Oral Biol..

[61] Patel M, Smith AJ, Sloan AJ, Smith G, Cooper PR (2009). Phenotype and behaviour of dental pulp cells during expansion culture. Arch Oral Biol..

[62] Coffey RJ, Leof EB, Shipley GD, Moses HL (1987). Suramin inhibition of growth factor receptor binding and mitogenicity in AKR-2B cells. J Cell Physiol..

[63] Ferrara N, Gerber HP, LeCouter J (2003). The biology of VEGF and its receptors. Nat Med..

[64] Muromachi K (2012). MMP-3 provokes CTGF/CCN2 production independently of protease activity and dependently on dynamin-related endocytosis, which contributes to human dental pulp cell migration. J Cell Biochem..

[65] Woo SM (2015). Vitamin D Promotes Odontogenic Differentiation of Human Dental Pulp Cells via ERK Activation. Mol Cells..

[66] Tran XV (2012). Effect of a calcium-silicate-based restorative cement on pulp repair. J Dent Res..

[67] Yoneda N (2017). Development of a root canal treatment model in the rat. Sci Rep..

[68] Kuremoto K (2014). Promotion of endodontic lesions in rats by a novel extraradicular biofilm model using obturation materials. Appl Environ Microbiol..

[69] Kiba W (2010). Efficacy of polyphasic calcium phosphates as a direct pulp capping material. J Dent..

[70] Shi S (2016). Comparison of *in vivo* dental pulp responses to capping with iRoot BP Plus and mineral trioxide aggregate. Int Endod J..

[71] Hu CC, Zhang C, Qian Q, Tatum NB (1988). Reparative dentin formation in rat molars after direct pulp capping with growth factors. J Endod..

[72] Six N, Lasfargues JJ, Goldberg M (2002). Differential repair responses in the coronal and radicular areas of the exposed rat molar pulp induced by recombinant human bone morphogenetic protein 7 (osteogenic protein 1). Arch Oral Biol..

[73] Hanada, K. *et al*. *In vitro* and *in vivo* effects of a novel bioactive glass-based cement used as a direct pulp capping agent. *J Biomed Mater Res B Appl Biomater*. Mar **25** (2018) 10.1002/jbm.b.34107 29575555

