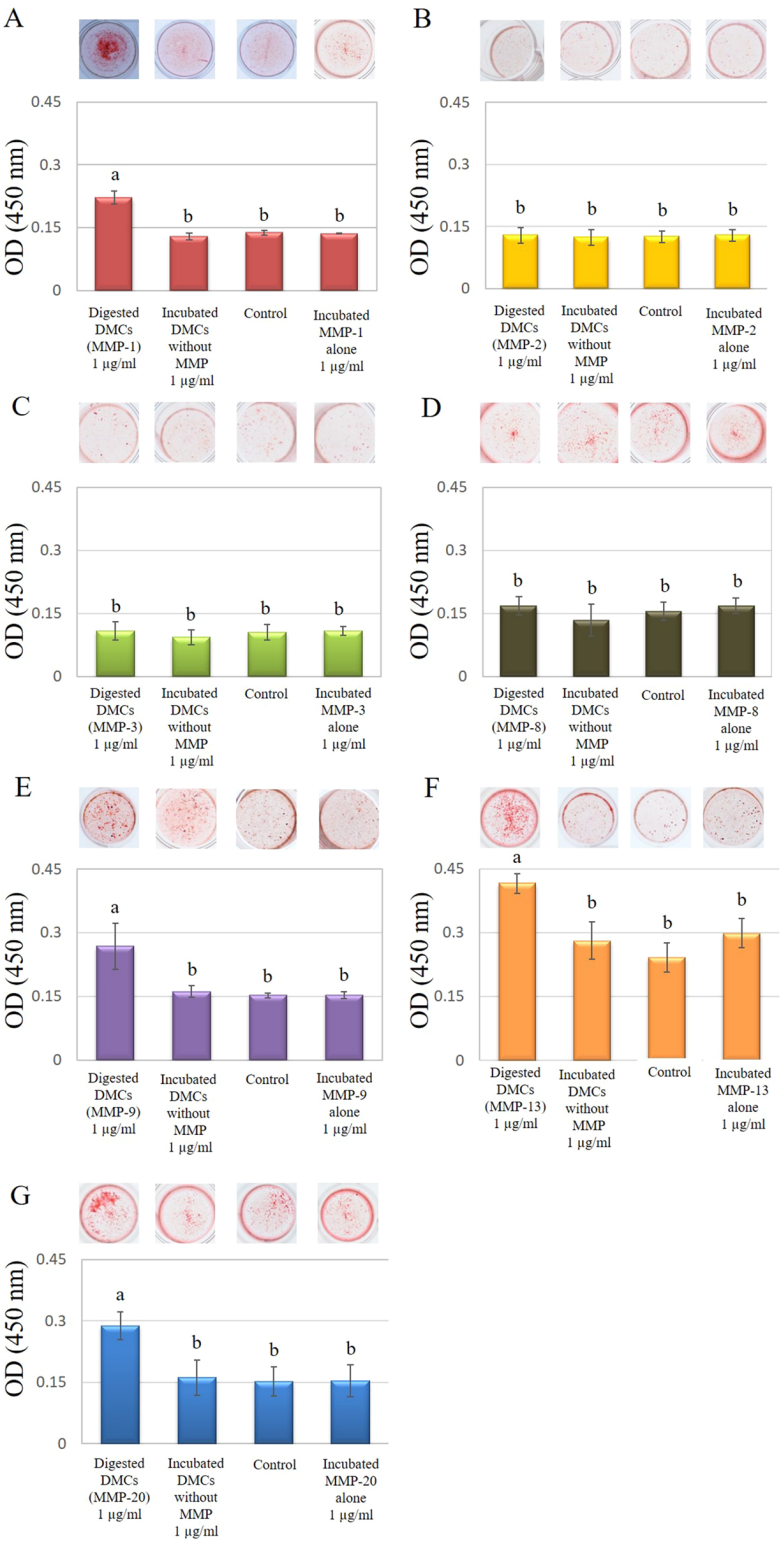# Author Correction: Dentinogenic effects of extracted dentin matrix components digested with matrix metalloproteinases

**DOI:** 10.1038/s41598-020-72251-9

**Published:** 2020-09-15

**Authors:** Motoki Okamoto, Yusuke Takahashi, Shungo Komichi, Paul R. Cooper, Mikako Hayashi

**Affiliations:** 1 Department of Restorative Dentistry and Endodontology, Osaka University Graduate School of Dentistry, Osaka, Japan; 2 Oral Biology, School of Dentistry, University of Birmingham, Birmingham, UK

Correction to: *Scientific Reports* 10.1038/s41598-018-29112-3, published online 16 July 2018

This Article contains errors in Figure 6. In assembling the figure, several incorrect images were incorporated, affecting the following: 6B, 1st panel; 6D, 4th panel; 6E, 2nd, 3rd, and 4th panel; 6G, 2nd panel. The correct figure appears below.