# Supplementary material for: Dentinogenic effects of extracted dentin matrix components digested with matrix metalloproteinases
Source: Sci Rep. 2018 Jul 16;8:10690. doi: 10.1038/s41598-018-29112-3 (PMC6048071; doi:10.1038/s41598-018-29112-3)
Supplement: Supplementary file 1 — Supplementary Figure [file 41598_2018_29112_MOESM1_ESM.pdf]

Dentinogenic effects of extracted dentin matrix components digested with matrix metalloproteinases

Motoki Okamoto, Yusuke Takahashi, Shungo Komichi, Paul R. Cooper, Mikako Hayashi

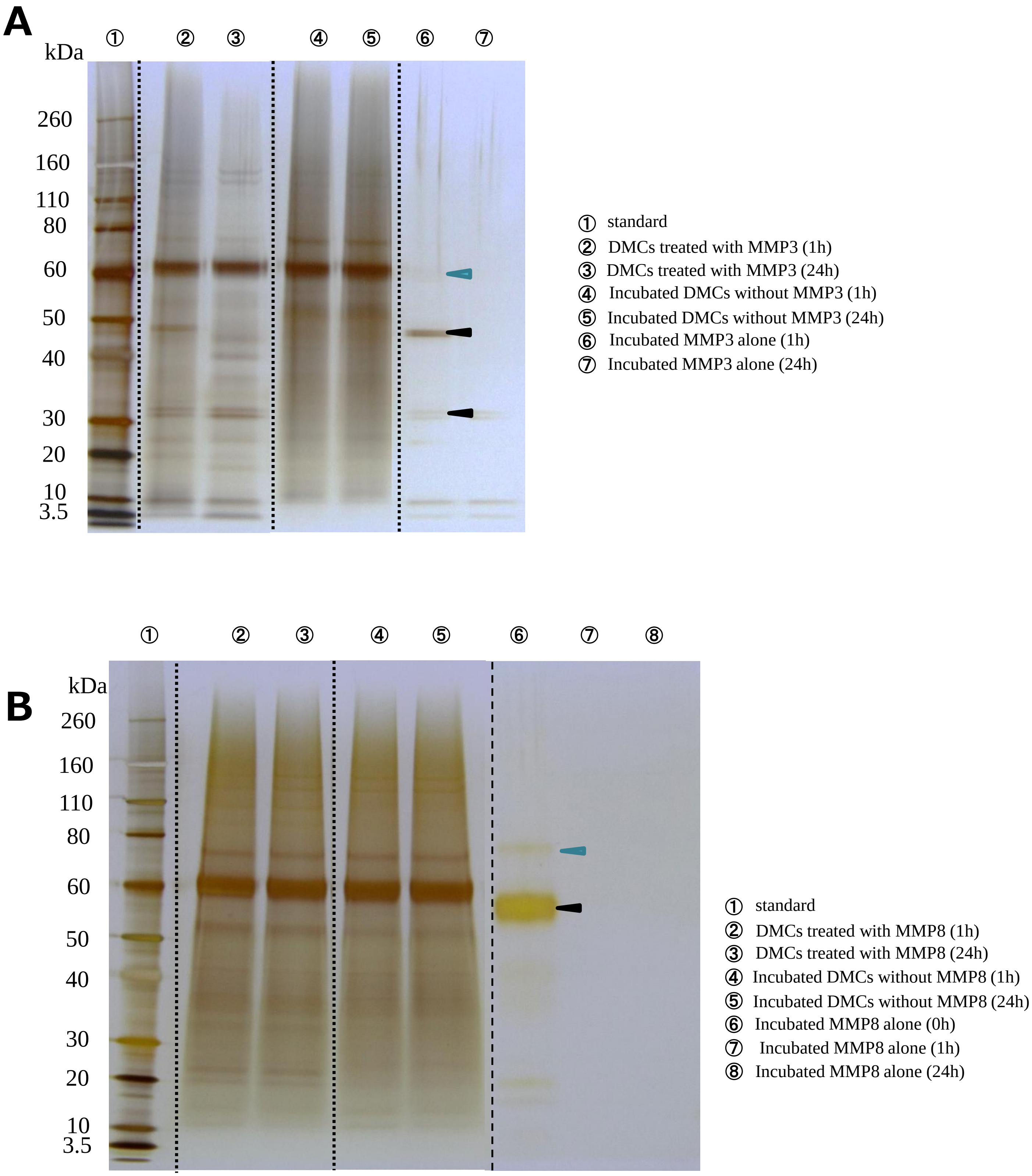

Supplementary figure 1

Protein profiles (silver staining) of DMCs treated with MMP-3 (A) or MMP-8 (B) are shown as a merged view by combining images of the cropped blots and gels. Dotted lines indicate the borders of the cropped blots. Dashed lines are shown on SDS-PAGE images to indicate lanes which were not originally electrophoresed together. Incubation time influenced the protein profile. Light blue arrowheads indicate predicted pro-MMP3 and pro-MMP8 bands. Black arrowheads indicate predicted active forms MMP3 and MMP8. Other low molecular bands may be degradation products. Original full blots/gels are presented in Supplementary Figures 14 and 16 (B).

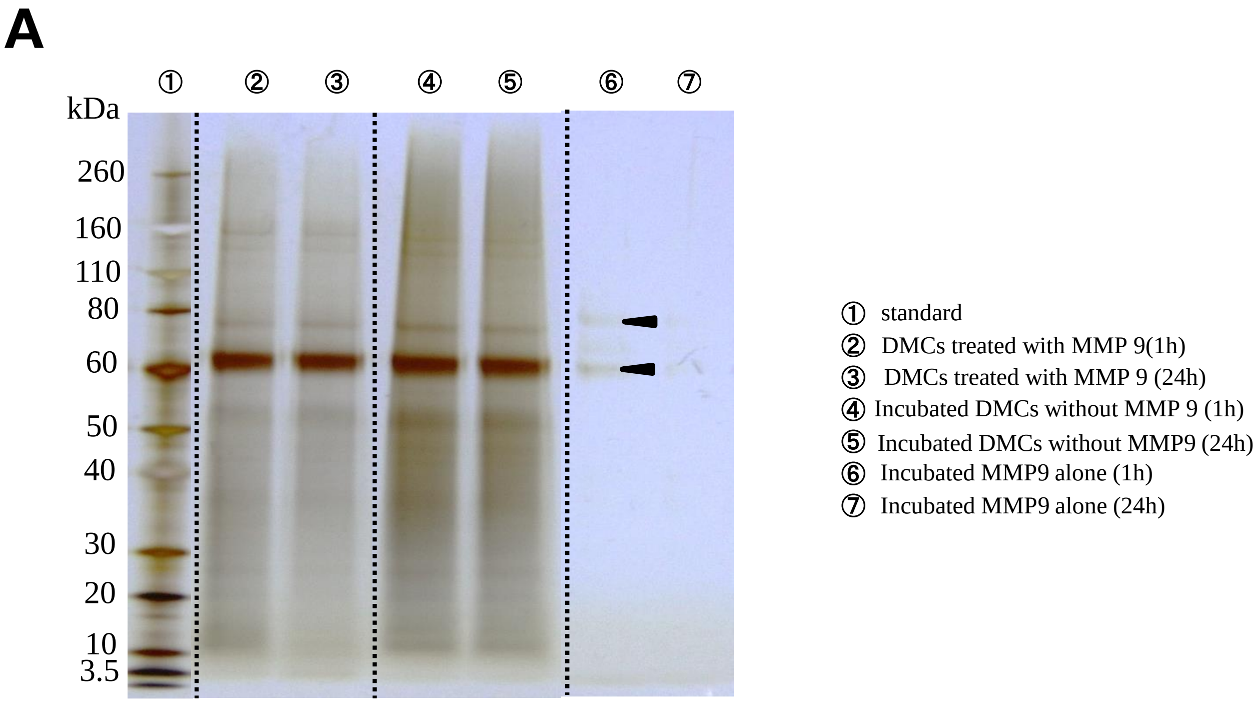

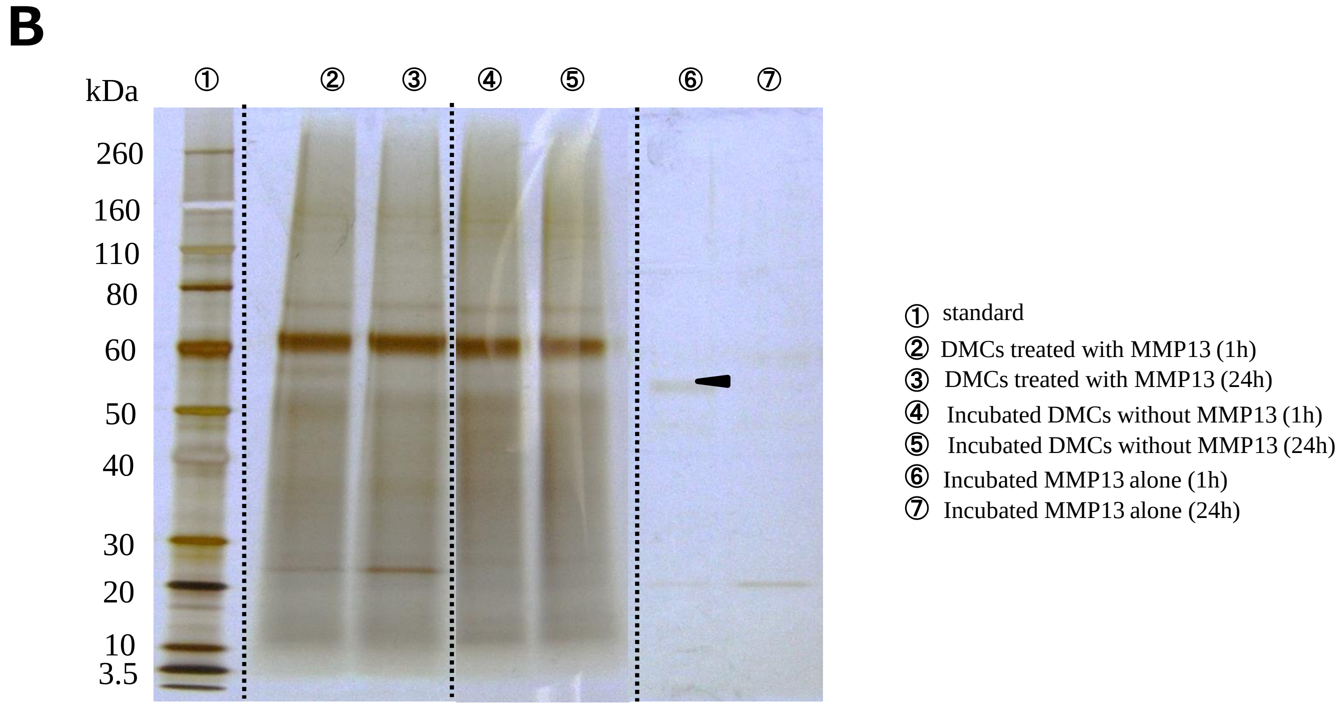

Supplementary figure 2

Protein profiles (silver staining) of DMCs treated with MMP-9 (A) or MMP-13 (B) were shown as the merged view by combining the cropped blots and gels. Dotted lines indicate the border of the cropped blots. The incubation time influenced the protein profile. Black arrowheads indicate active form of predicted MMP9 and MMP13 respectively. Other low molecular bands may represent degradation products. Pro form of predicted MMP 9 and MMP13 bands could not be detected. Original blot/gel images are presented in Supplementary Figure 15.

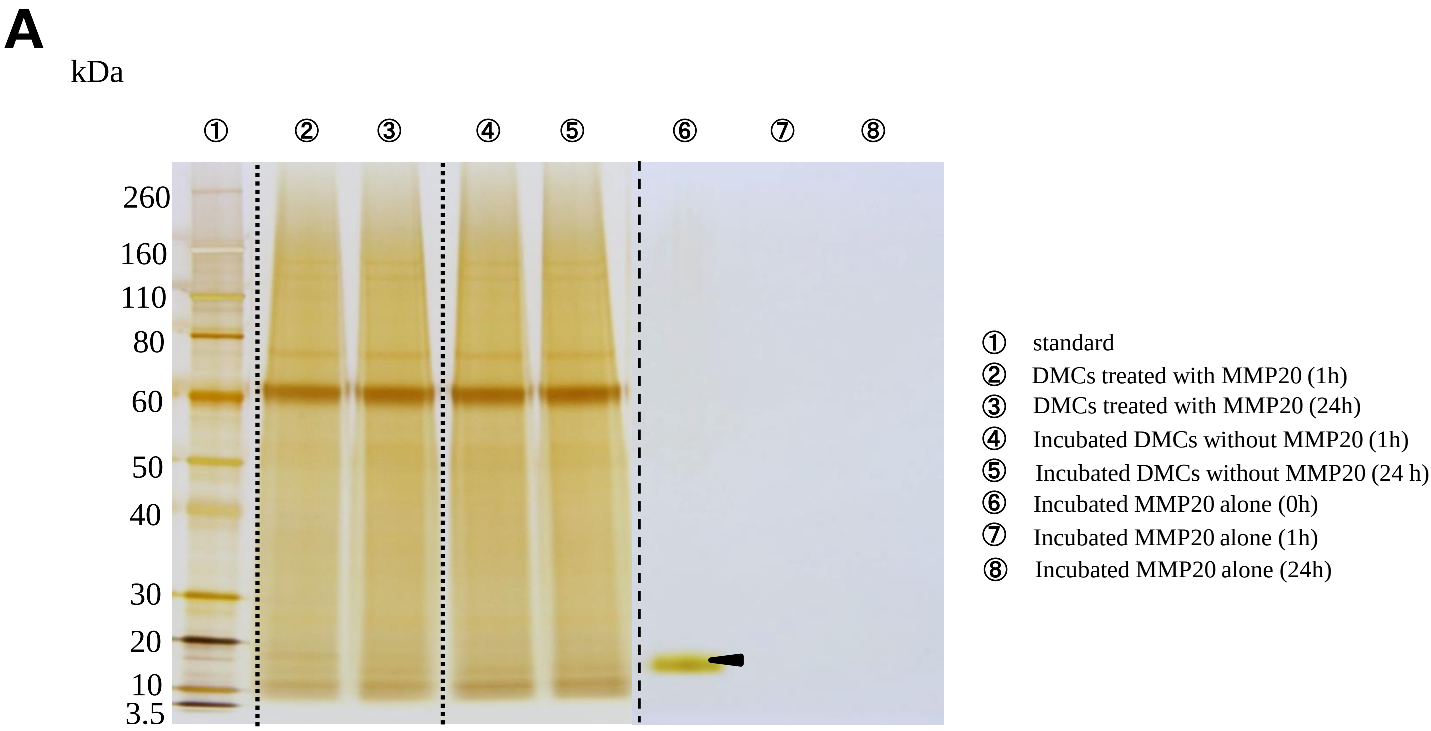

Supplementary figure 3

Protein profiles (silver staining) of DMCs treated with MMP-20 (A) are shown as a merged view by combining the cropped blots and gels. Dotted lines indicate the border of the cropped blots. Dashed lines are shown on SDS-PAGE images to indicate lanes which were not originally electrophoresed together. The incubation time influenced the protein profile. Black arrowhead indicates predicted MMP20. Pro form of predicted MMP 20 bands could not be detected. Original blots/gel images are presented in Supplementary Figure 16 (A).

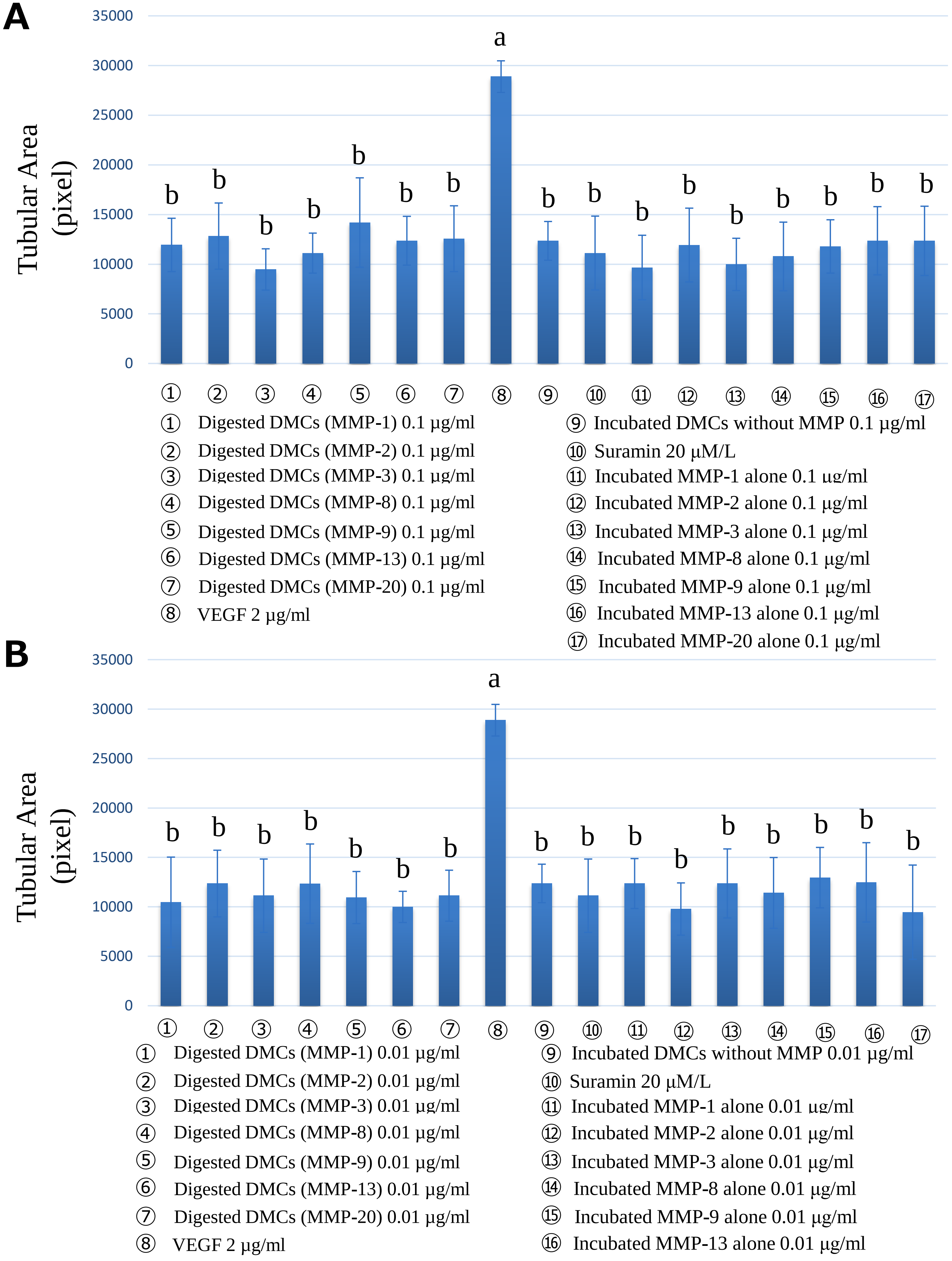

Supplementary figure 4

Quantification of tubule formation. No significant differences were observed with lower concentrations (0.01- 0.1 µg/ml) of DMCs treated with the above described MMPs. Groups with similar lower case letters (i.e., a and b) are not significantly different. Data represent three independent experiments.

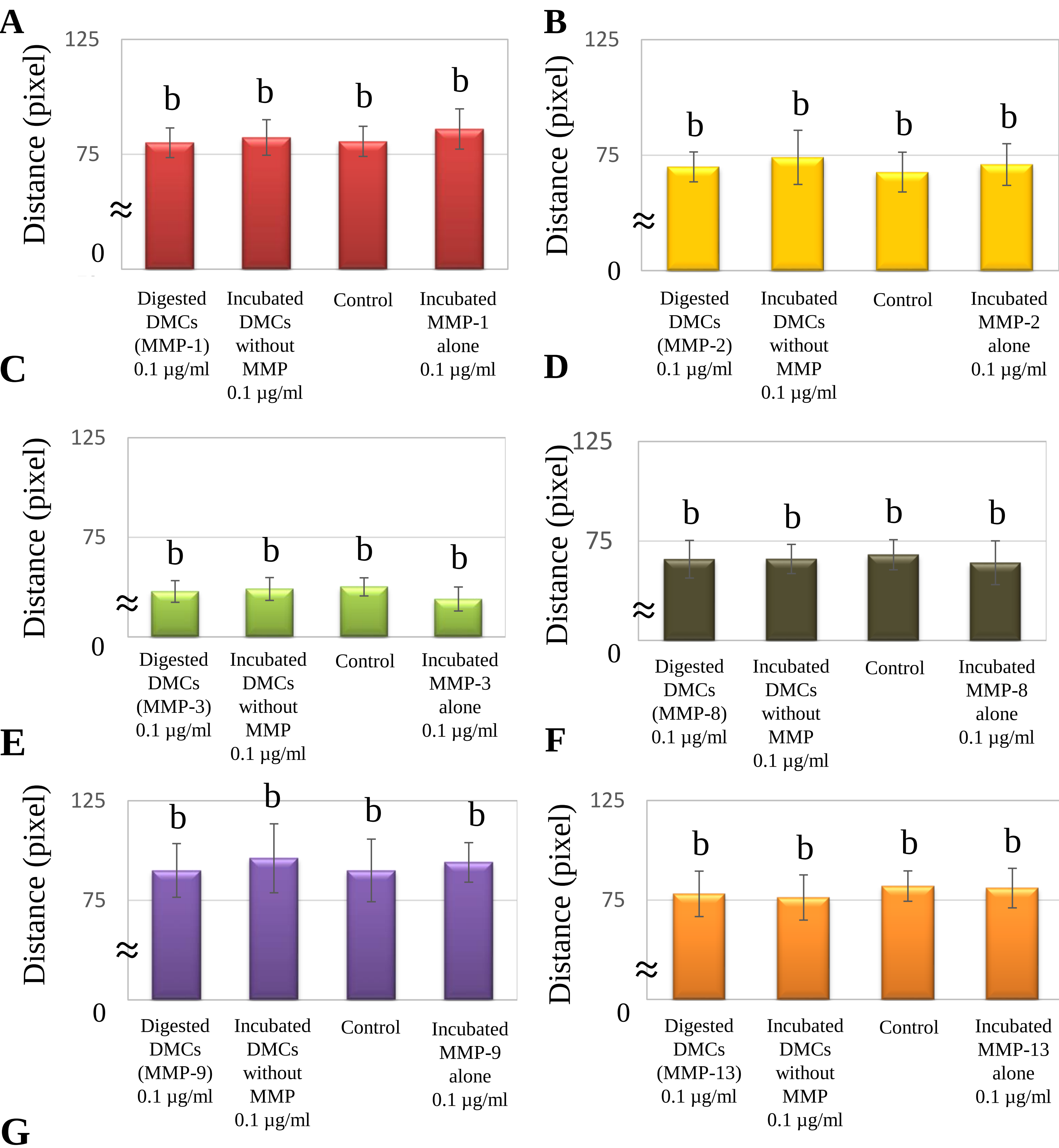

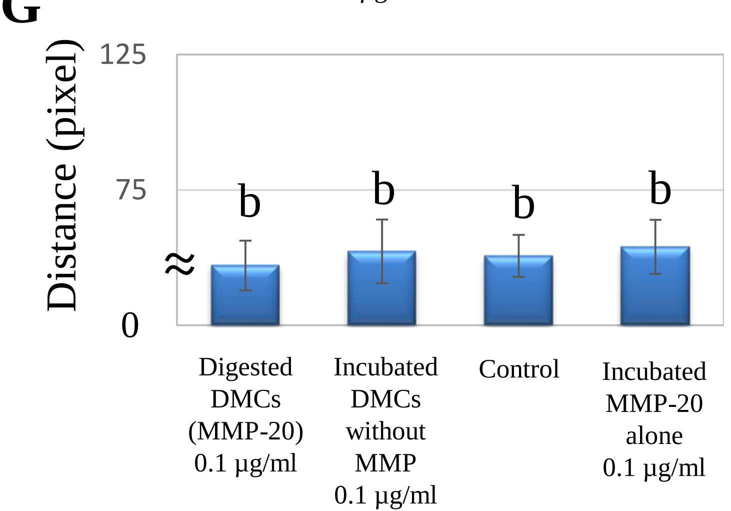

Supplementary figure 5

Effects of 0.1 and 0.01 μg/ml of DMCs treated with MMPs on wound scratch (A-G, H-N) and chemotactic (O, P) cell migration. Groups with similar lower case letters (i.e., a and b) are not significantly different. Data represent five independent experiments.

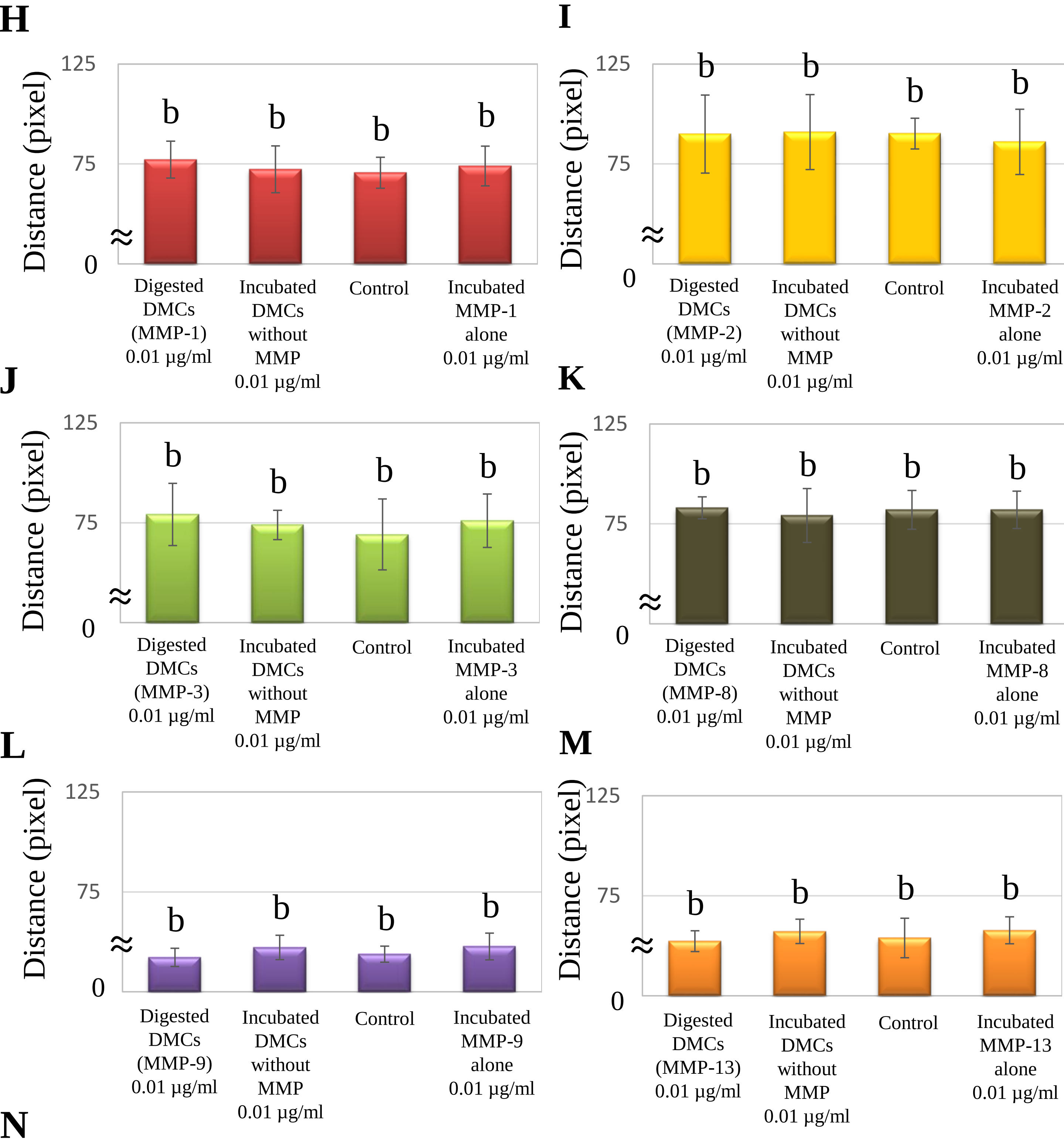

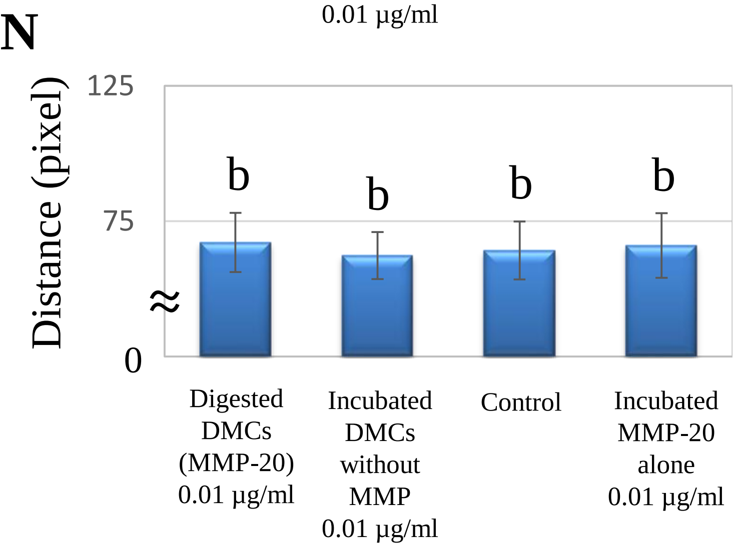

Supplementary figure 5

O

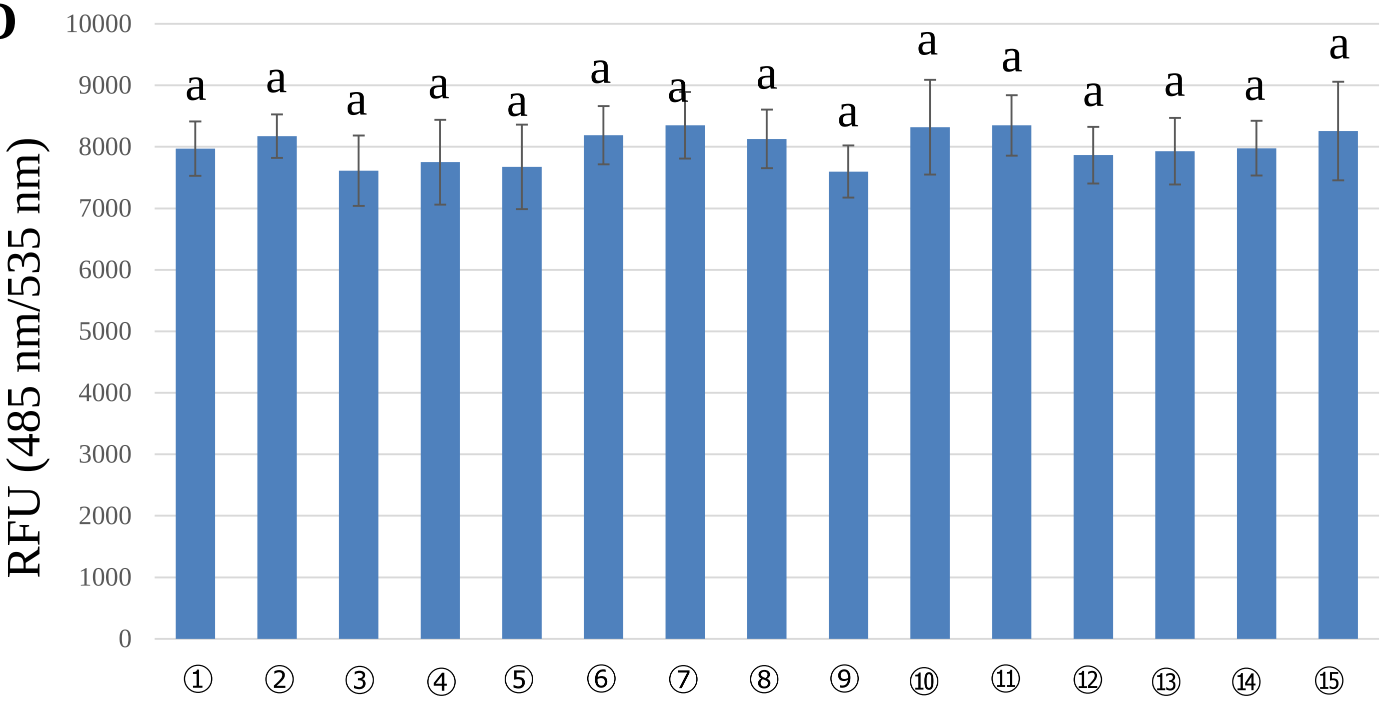

① Digested DMCs (MMP-1) 0.1 μg/ml
② Digested DMCs (MMP-2) 0.1 μg/ml
③ Digested DMCs (MMP-3) 0.1 μg/ml
④ Digested DMCs (MMP-8) 0.1 μg/ml
⑤ Digested DMCs (MMP-9) 0.1 μg/ml
⑥ Digested DMCs(MMP-13) 0.1 μg/ml
⑦ Digested DMCs(MMP-20) 0.1 μg/ml
⑧ Incubated DMCs without MMP 0.1 μg/ml
⑨ Incubated MMP-1 alone 0.1 μg/ml
⑩ Incubated MMP-2 alone 0.1 μg/ml
⑪ Incubated MMP-3 alone 0.1 μg/ml
⑫ Incubated MMP-8 alone 0.1 μg/ml
⑬ Incubated MMP-9 alone 0.1 μg/ml
⑭ Incubated MMP-13 alone 0.1 μg/ml
⑮ Incubated MMP-20 alone 0.1 μg/ml

P

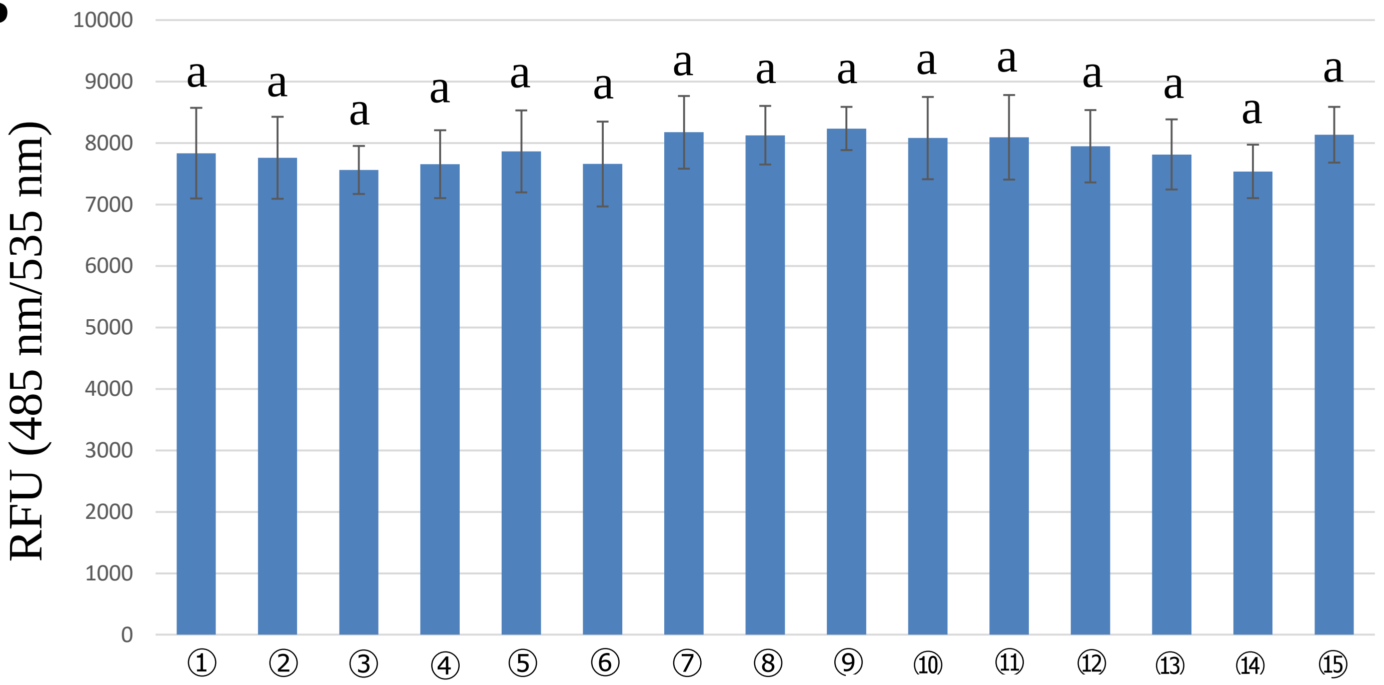

① Digested DMCs (MMP-1) 0.01 μg/ml
② Digested DMCs (MMP-2) 0.01 μg/ml
③ Digested DMCs (MMP-3) 0.01 μg/ml
④ Digested DMCs (MMP-8) 0.01 μg/ml
⑤ Digested DMCs (MMP-9) 0.01 μg/ml
⑥ Digested DMCs(MMP-13) 0.01 μg/ml
⑦ Digested DMCs(MMP-20) 0.01 μg/ml
⑧ Incubated DMCs without MMP 0.01 μg/ml
⑨ Incubated MMP-1 alone 0.01 μg/ml
⑩ Incubated MMP-2 alone 0.01 μg/ml
⑪ Incubated MMP-3 alone 0.01 μg/ml
⑫ Incubated MMP-8 alone 0.01 μg/ml
⑬ Incubated MMP-9 alone 0.01 μg/ml
⑭ Incubated MMP-13 alone 0.01 μg/ml
⑮ Incubated MMP-20 alone 0.01 μg/ml

Supplementary figure 5

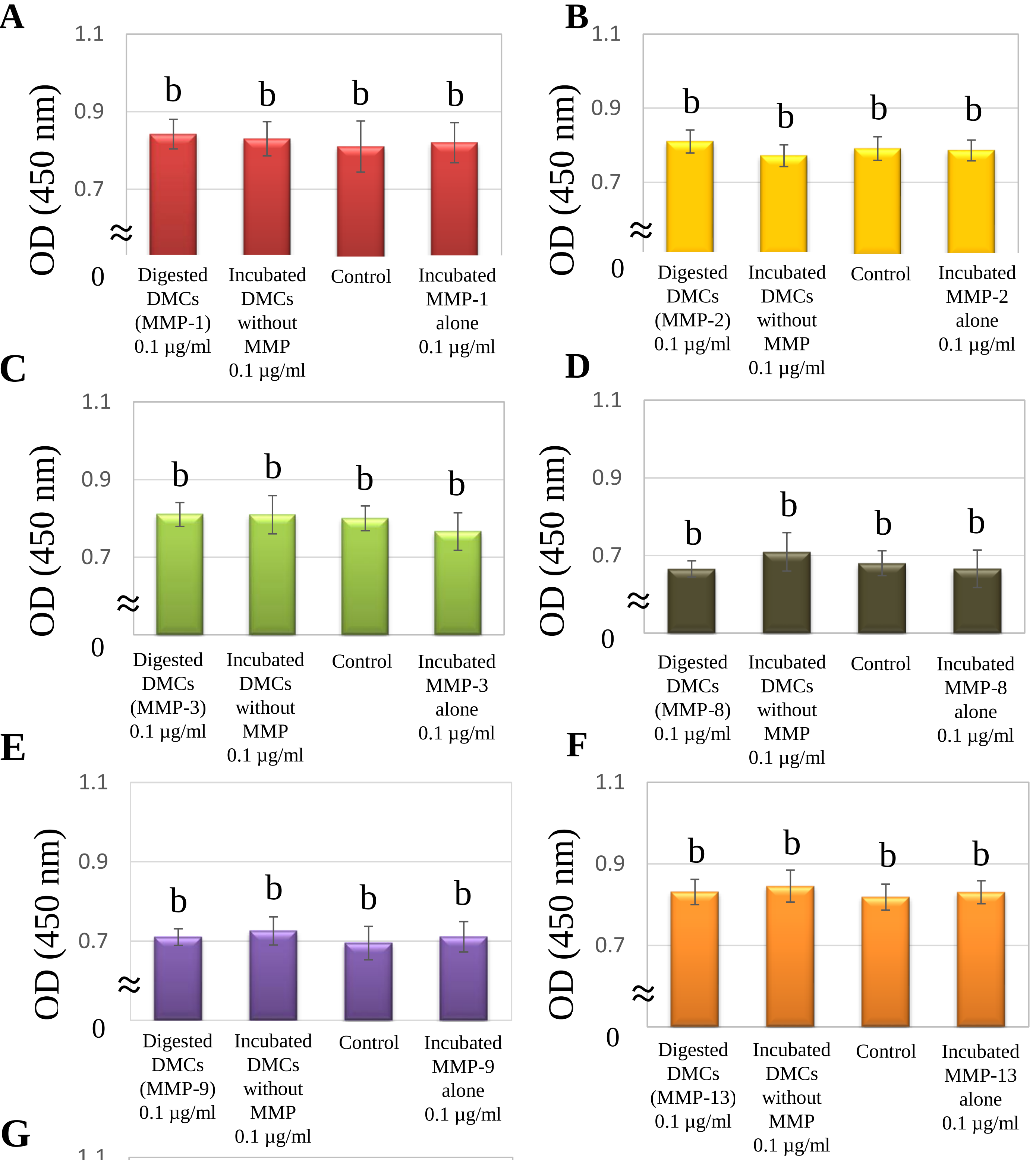

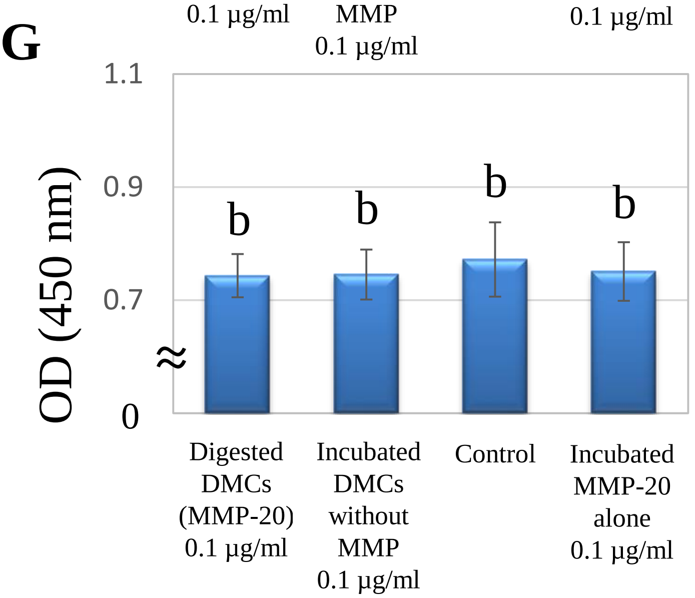

Supplementary figure 6

Effects of 0.1 and 0.01 µg/ml of DMCs treated with MMP-1 (A, H), MMP-2 (B, I), MMP-3 (C, J), MMP-8 (D, K), MMP-9 (E, L), MMP-13 (F, M) and MMP-20 (G, N) on cell proliferation of rat primary pulp cells. Groups with similar lower case letters (i.e., a and b) are not significantly different. Data represent five independent experiments.

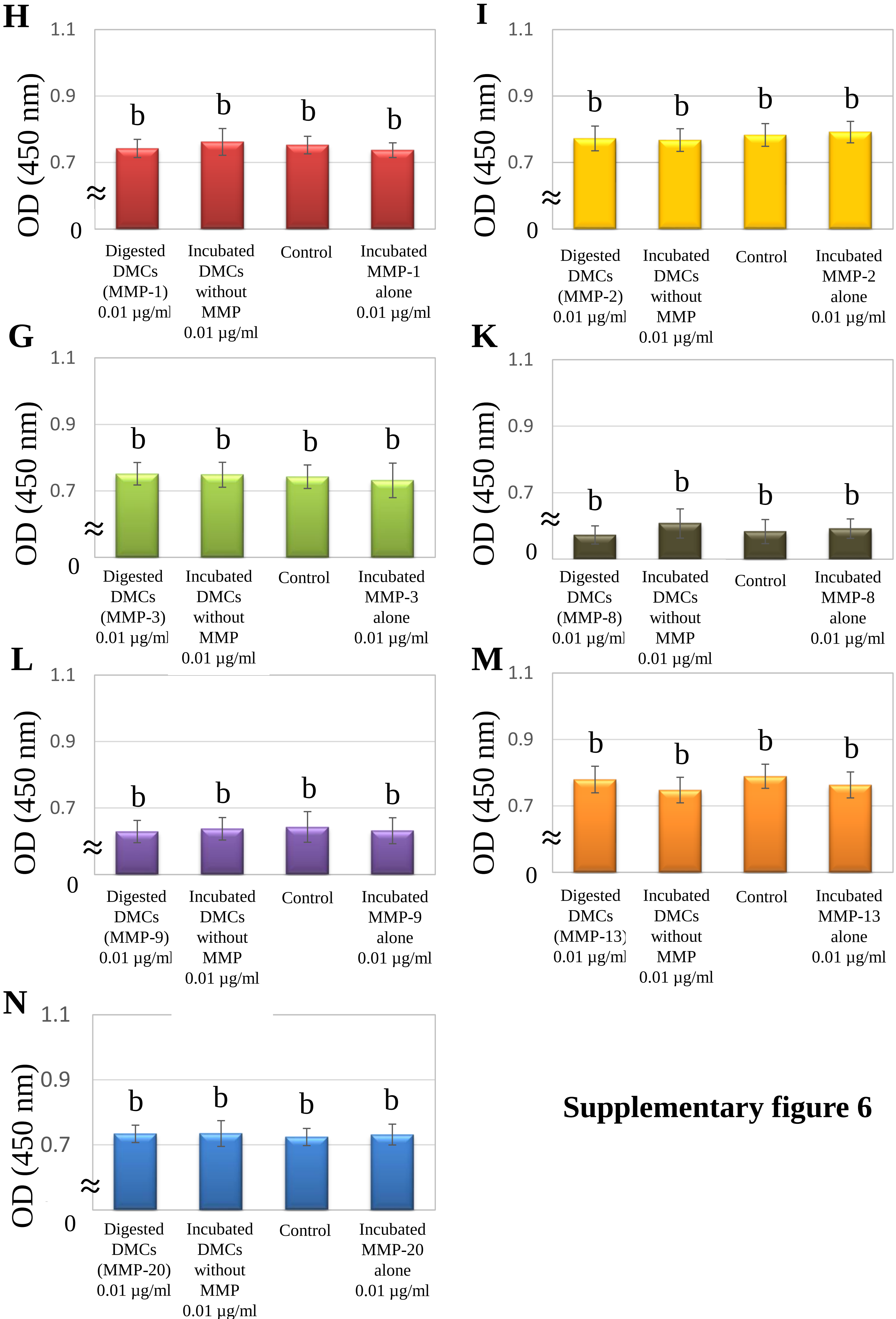

Supplementary figure 6

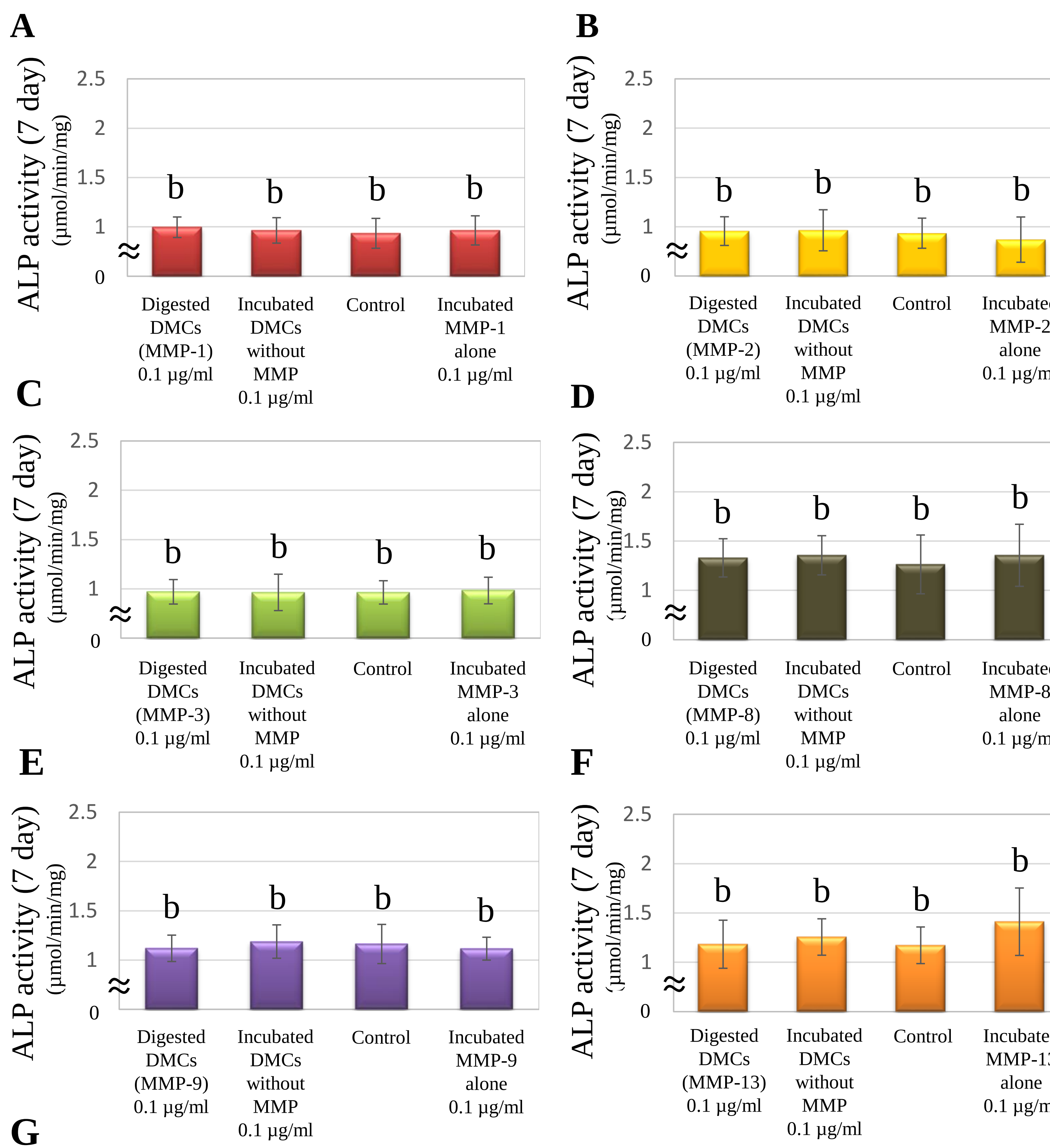

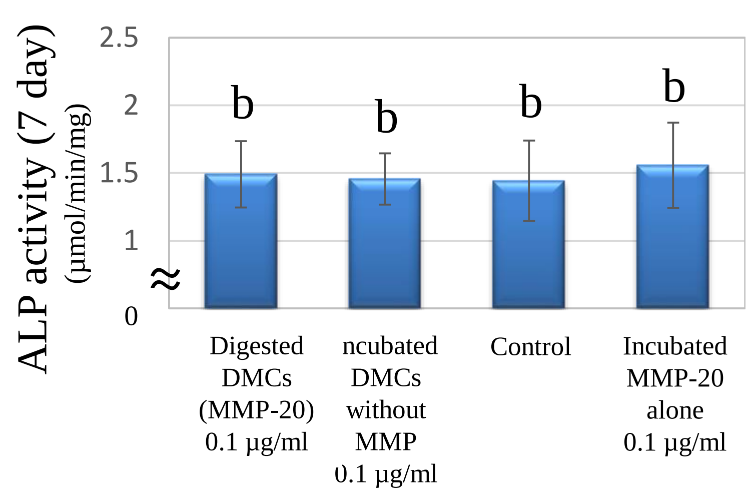

Supplementary figure 7

Effects of 0.1 and 0.01 μg/ml of MMP-treated DMCs on cell differentiation in rat primary pulp cells. Cells were treated with DMCs treated with MMP-1 (A, H), MMP-2 (B, I), MMP-3 (C, J), MMP-8 (D, K), MMP-9 (D, L), MMP-13 (E, M), and MMP-20 (G, N), incubated DMCs without MMP or medium alone containing 50 μg/ml of ascorbic acid and 10 mM of β-glycerophosphate for 7 days.

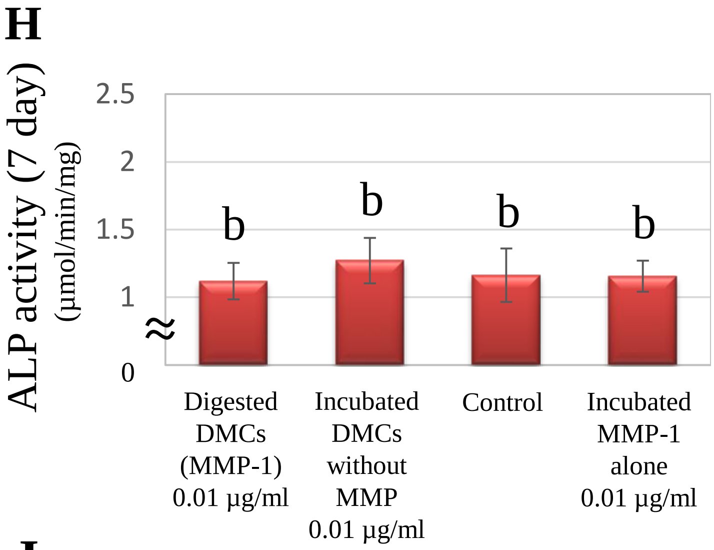

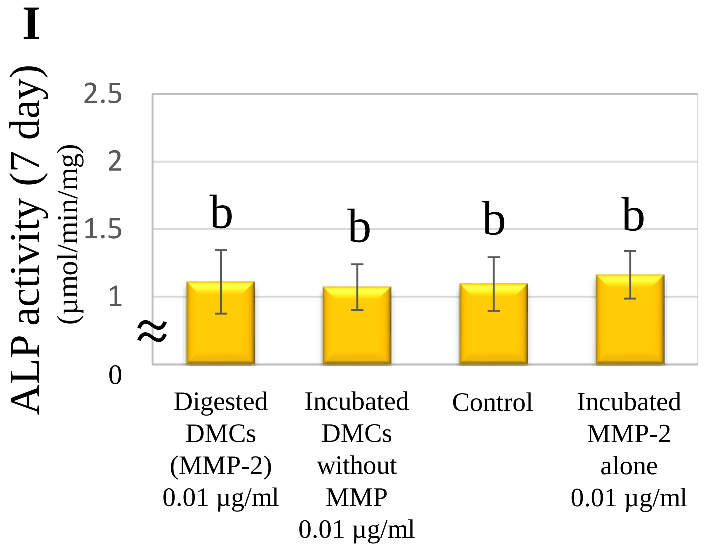

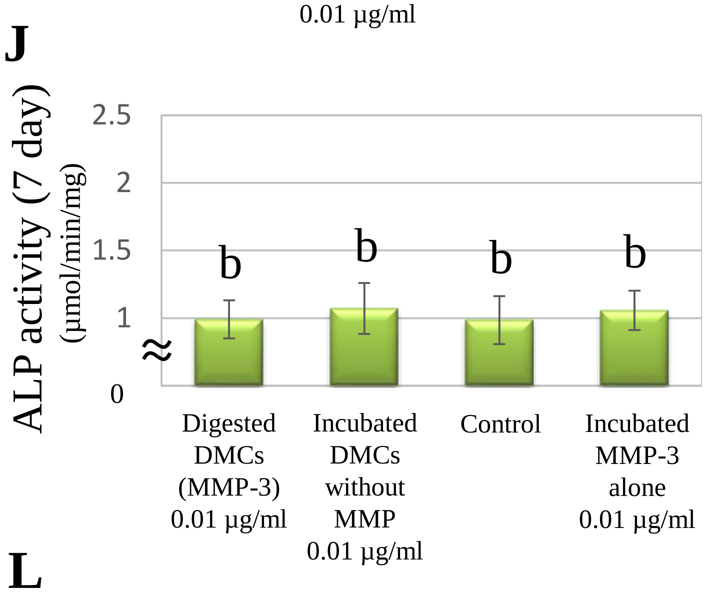

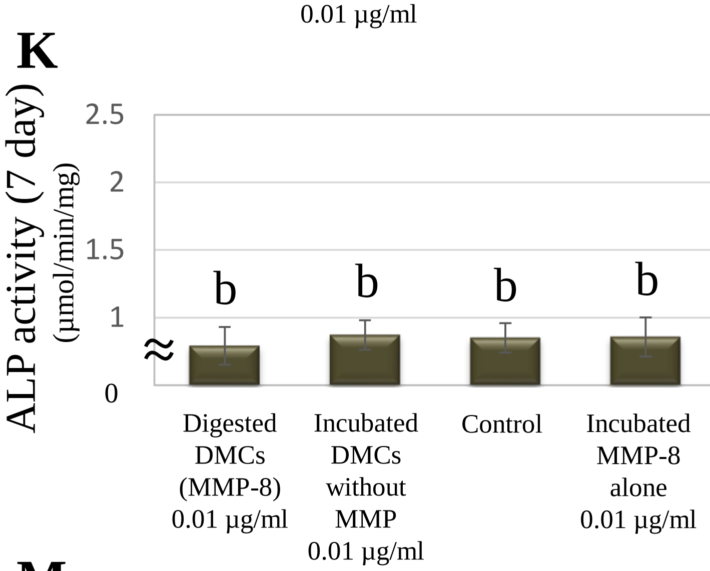

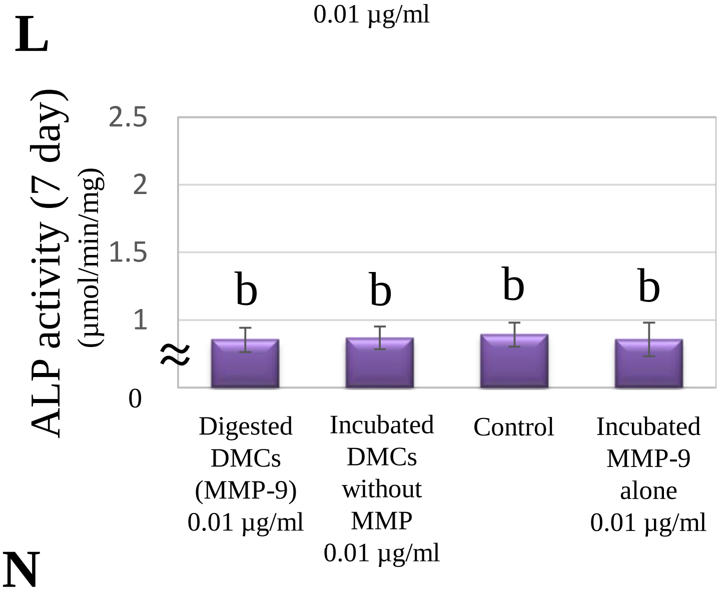

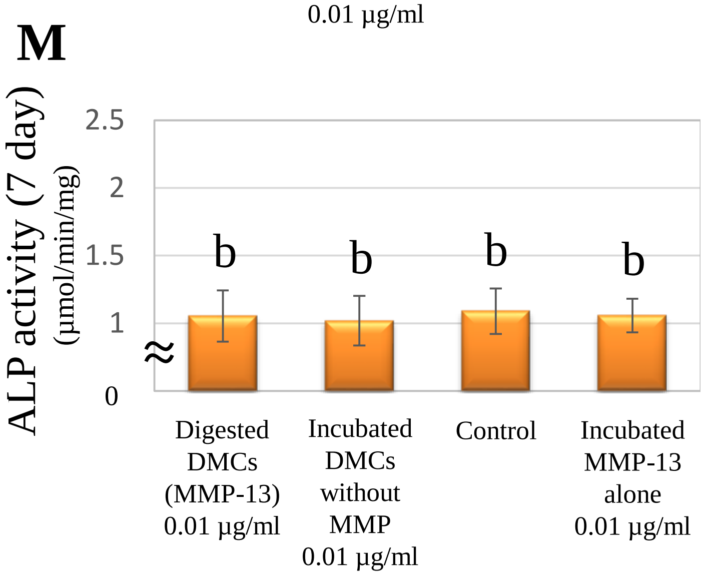

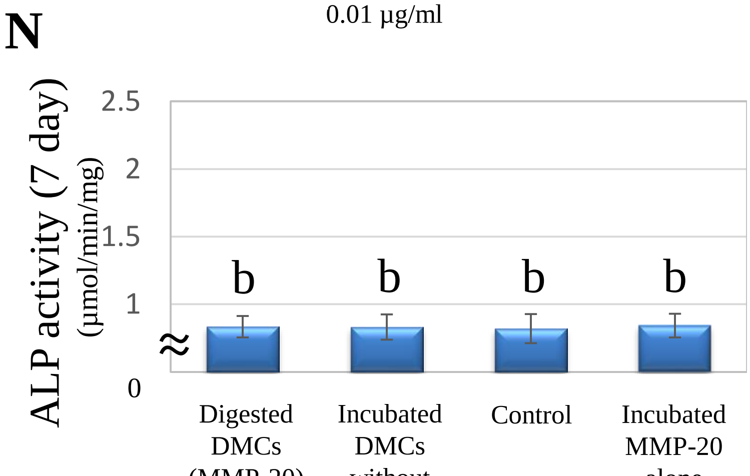

Supplementary figure 7

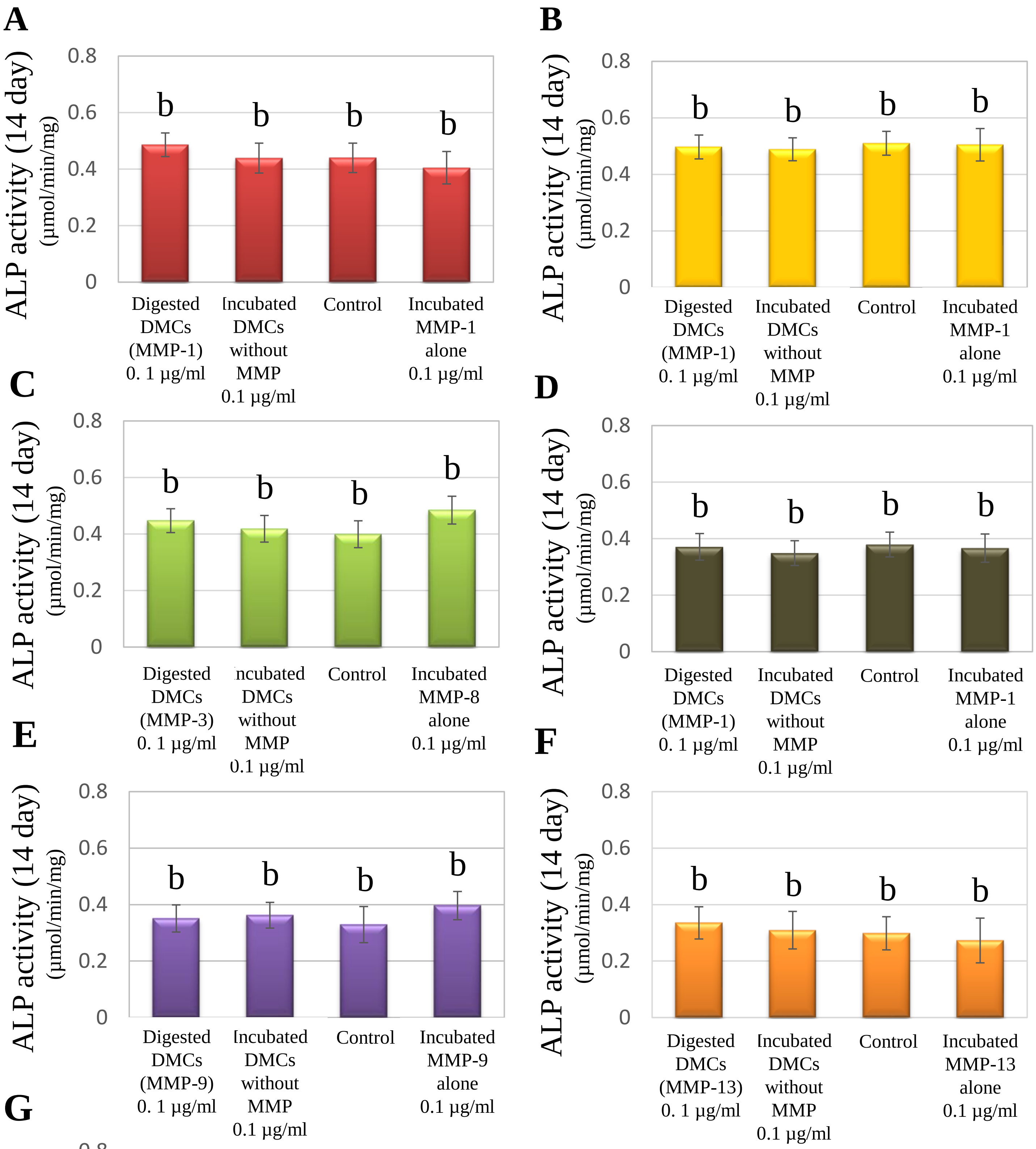

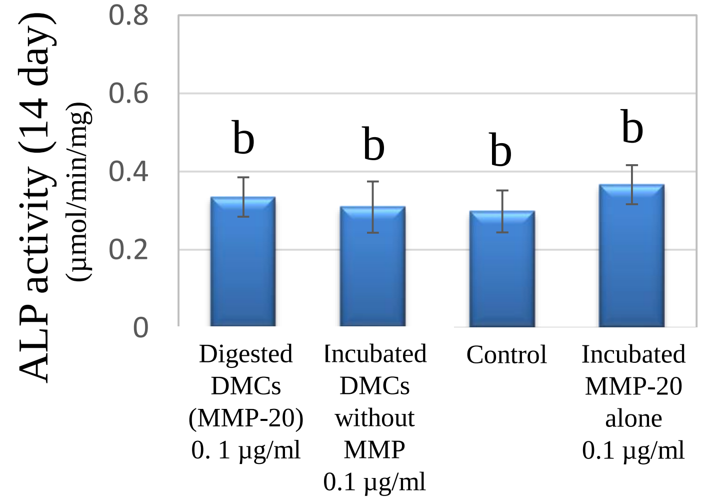

Supplementary figure 8

Effects of 0.1 and 0.01 μg/ml of MMP-treated DMCs on cell differentiation in rat primary pulp cells. Cells were treated with DMCs treated with MMP-1 (A, H), MMP-2 (B, I), MMP-3 (C, J), MMP-8 (D, K), MMP-9 (D, L), MMP-13 (E, M), and MMP-20 (G, N), incubated DMCs without MMP or medium alone containing 50 μg/ml of ascorbic acid and 10 mM of β-glycerophosphate for 14 days.

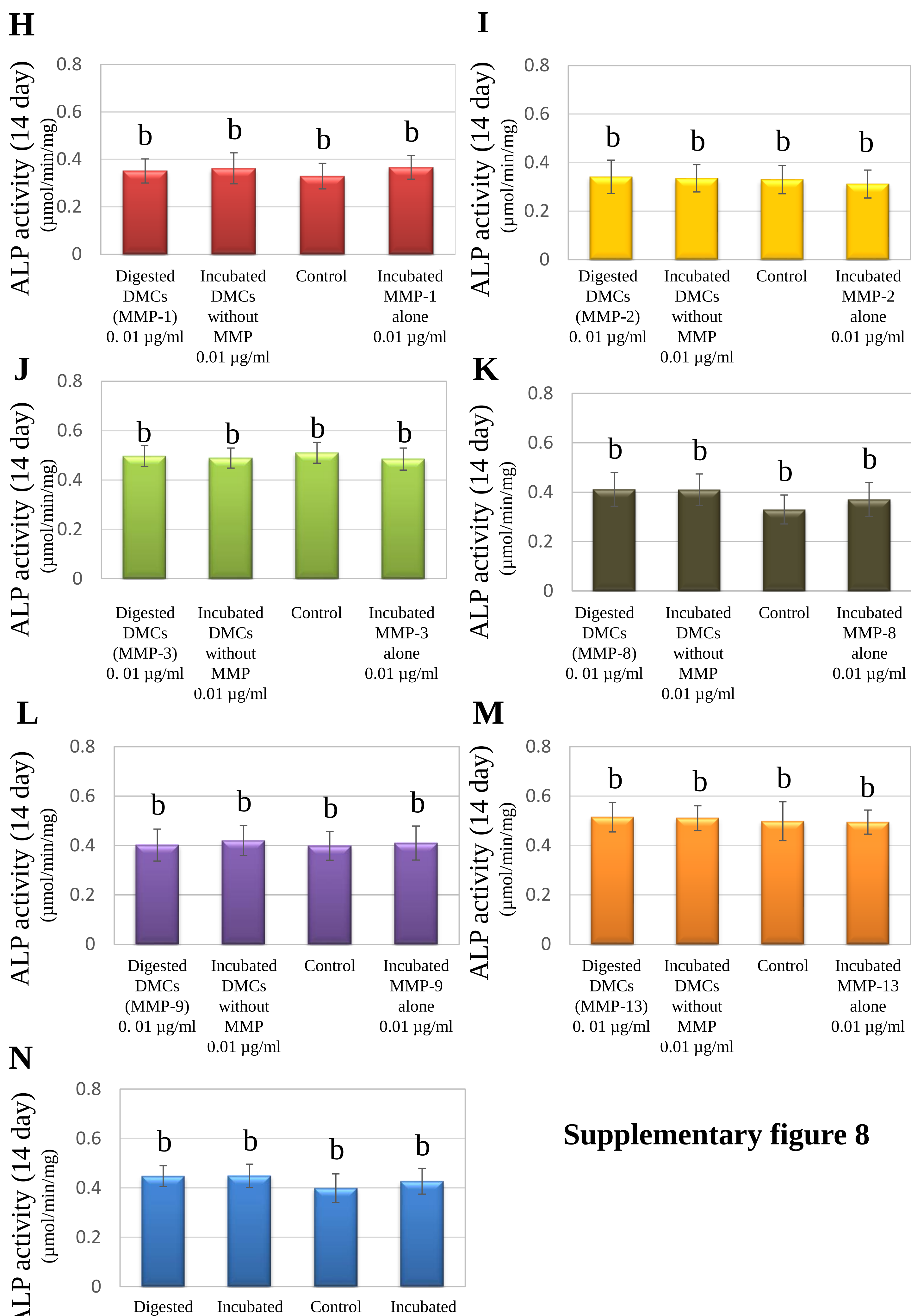

Supplementary figure 8

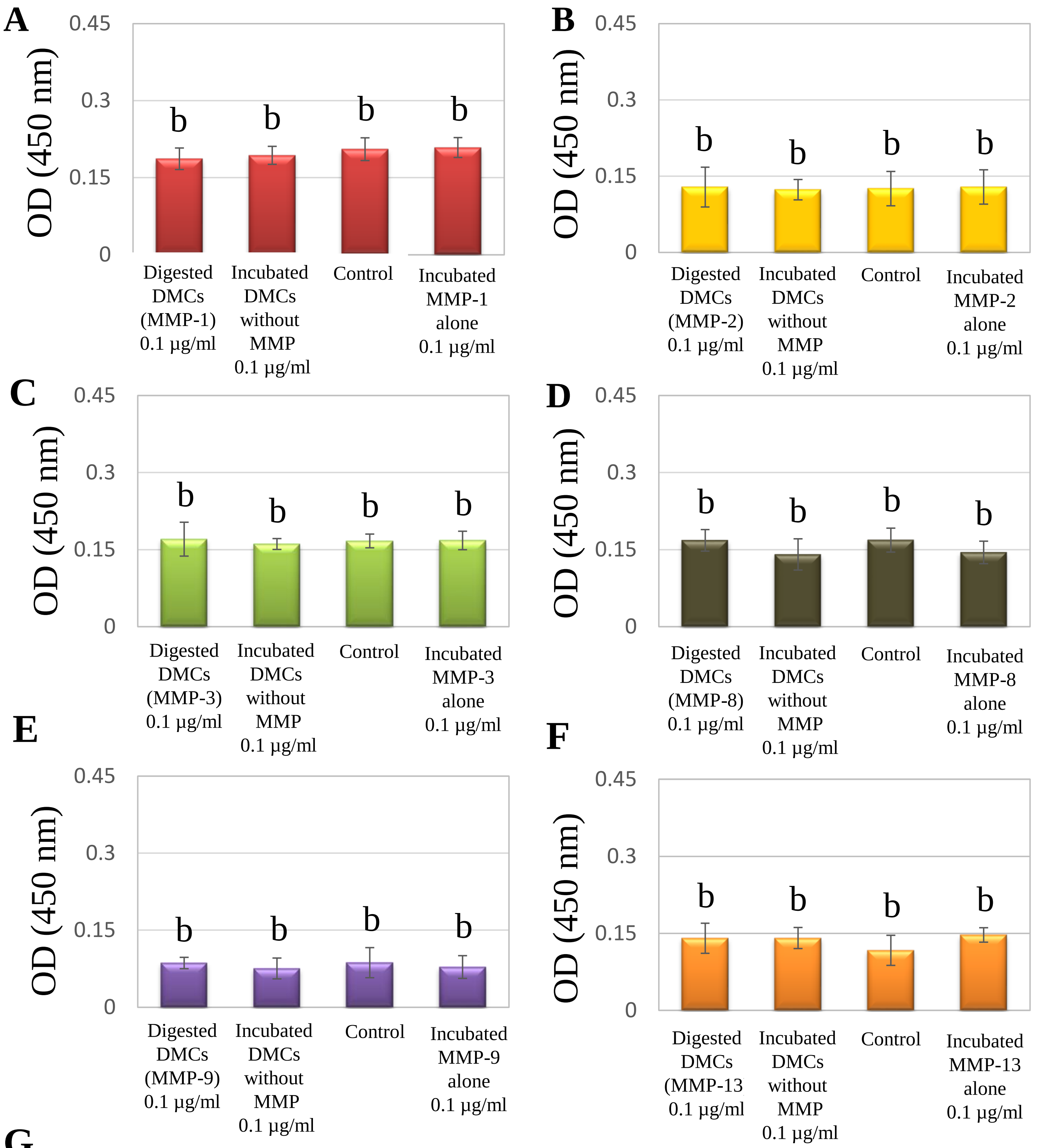

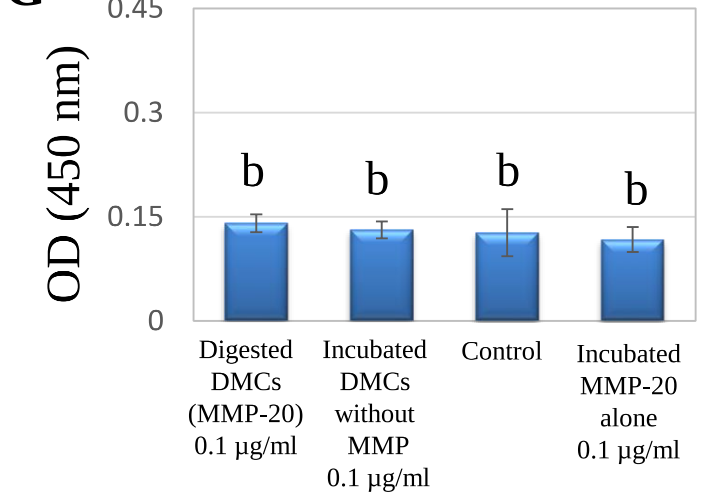

Supplementary figure 9

Effects of 0.1 and 0.01µg/ml of DMCs treated with MMP-1 (A, H), MMP-2 (B, I), MMP-3 (C, J), MMP-8 (D, K), MMP-9 (E, L), MMP-13 (F, M), or MMP-20 (G, N). Groups with similar lower case letters (i.e., a and b) are not significantly different. Data represent five independent experiments.

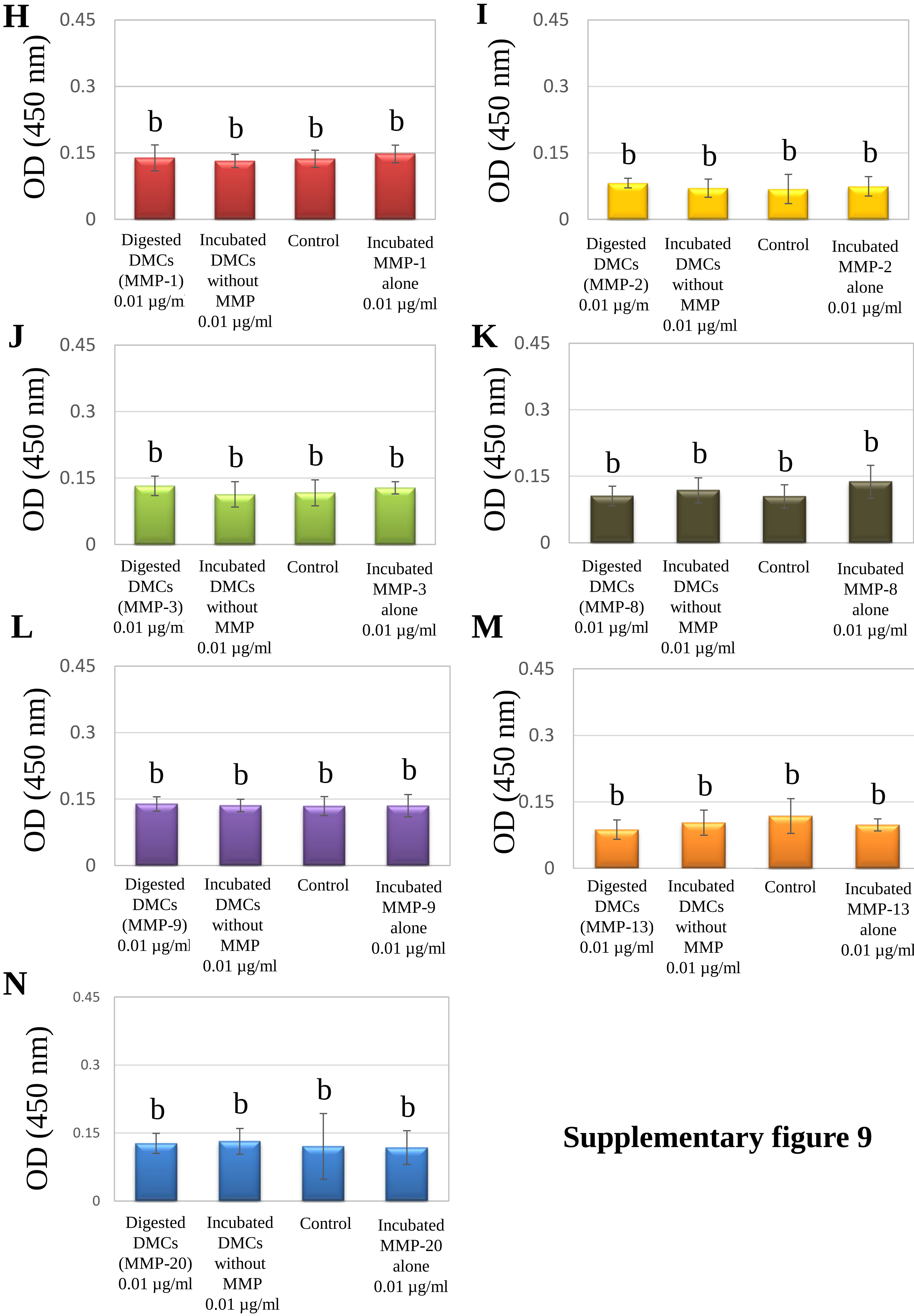

Supplementary figure 9

A

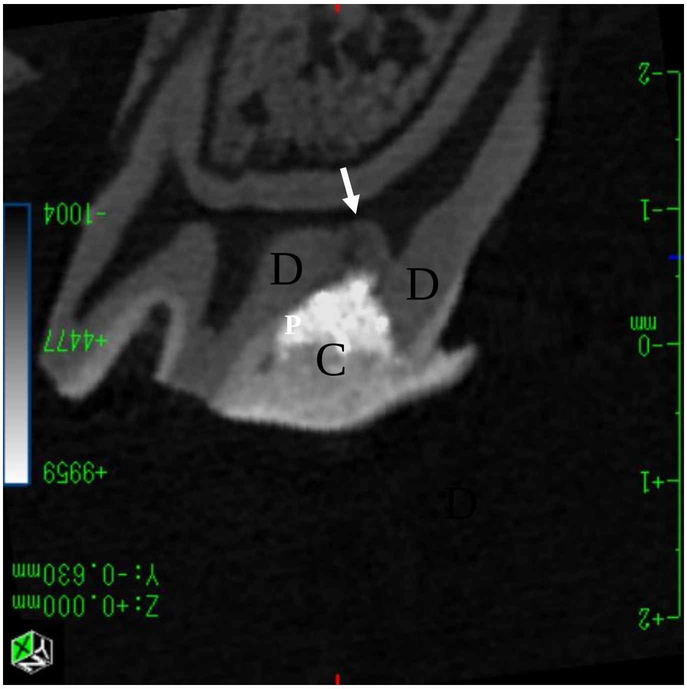

B

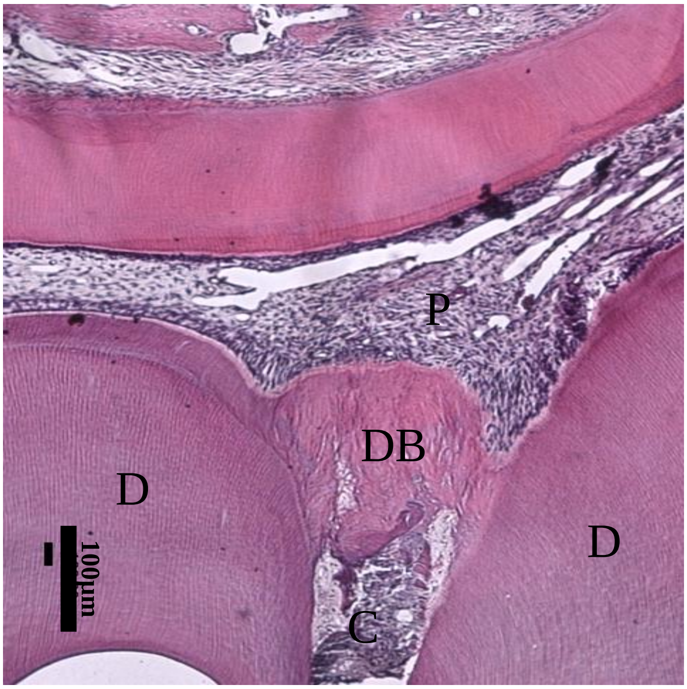

Supplementary figure 10

Micro-CT image (A) and histological image (B) (100×) of tertiary dentin induced ProRoot MTA 28 days after pulp capping. C=cavity, D=dentin, DB=dentin bridge, P=pulp, White arrow=dentin bridge.

A

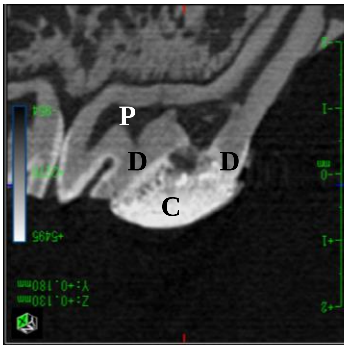

Incubated MMP-1
alone 1 μg/ml

B

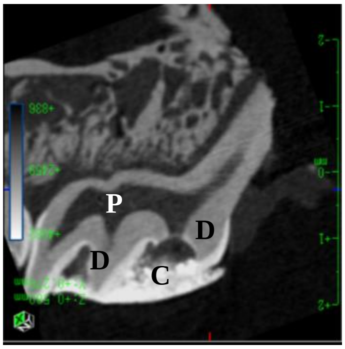

Incubated MMP-2
alone 1 μg/ml

C

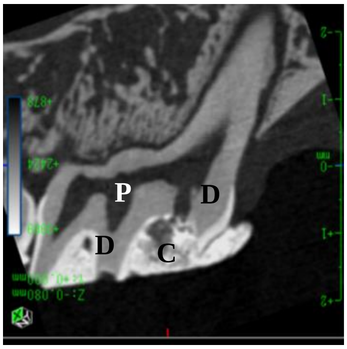

Incubated MMP-3
alone 1 μg/ml

D

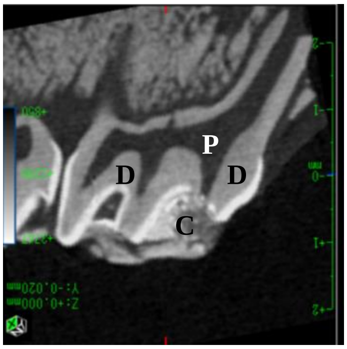

Incubated MMP-8
alone 1 μg/ml

E

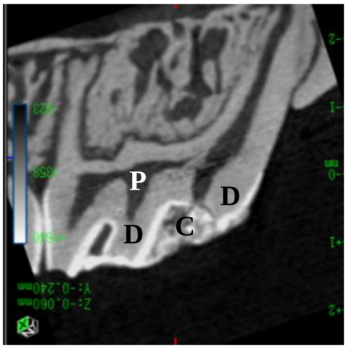

Incubated MMP-9
alone 1 μg/ml

F

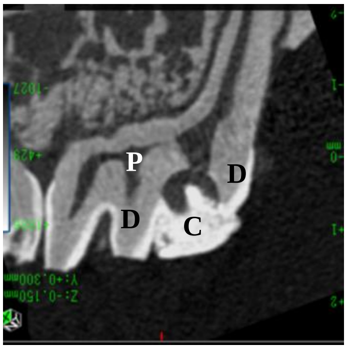

Incubated MMP-13
alone 1 μg/ml

G

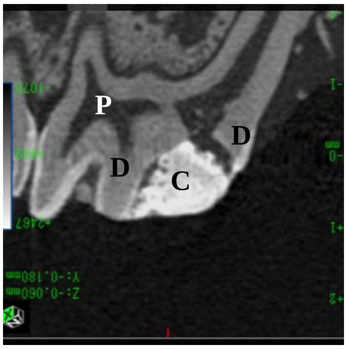

Incubated MMP-20
alone 1 μg/ml

I

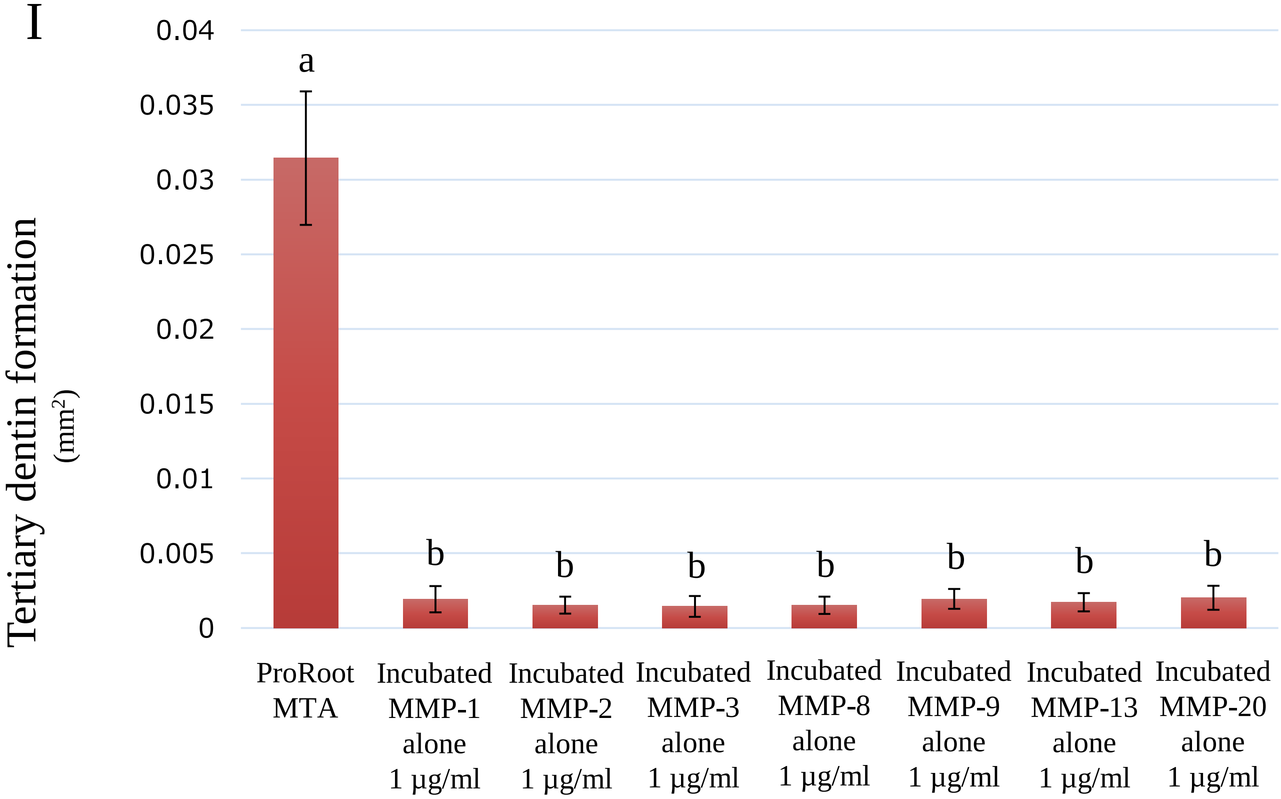

Supplementary figure 11

Micro CT images of tertiary dentin formation 28 days after direct pulp capping using 1 μg/ml of incubated MMP-1 (A), MMP-2 (B), MMP-3 (C), MMP-8 (D), MMP-9 (E), MMP-13 (F) and MMP-20 (G). The tertiary dentin area 28 days after direct pulp capping using ProRoot MTA, incubated MMP-1, -2, -3, -8, -9, -13 and -20 alone (I).

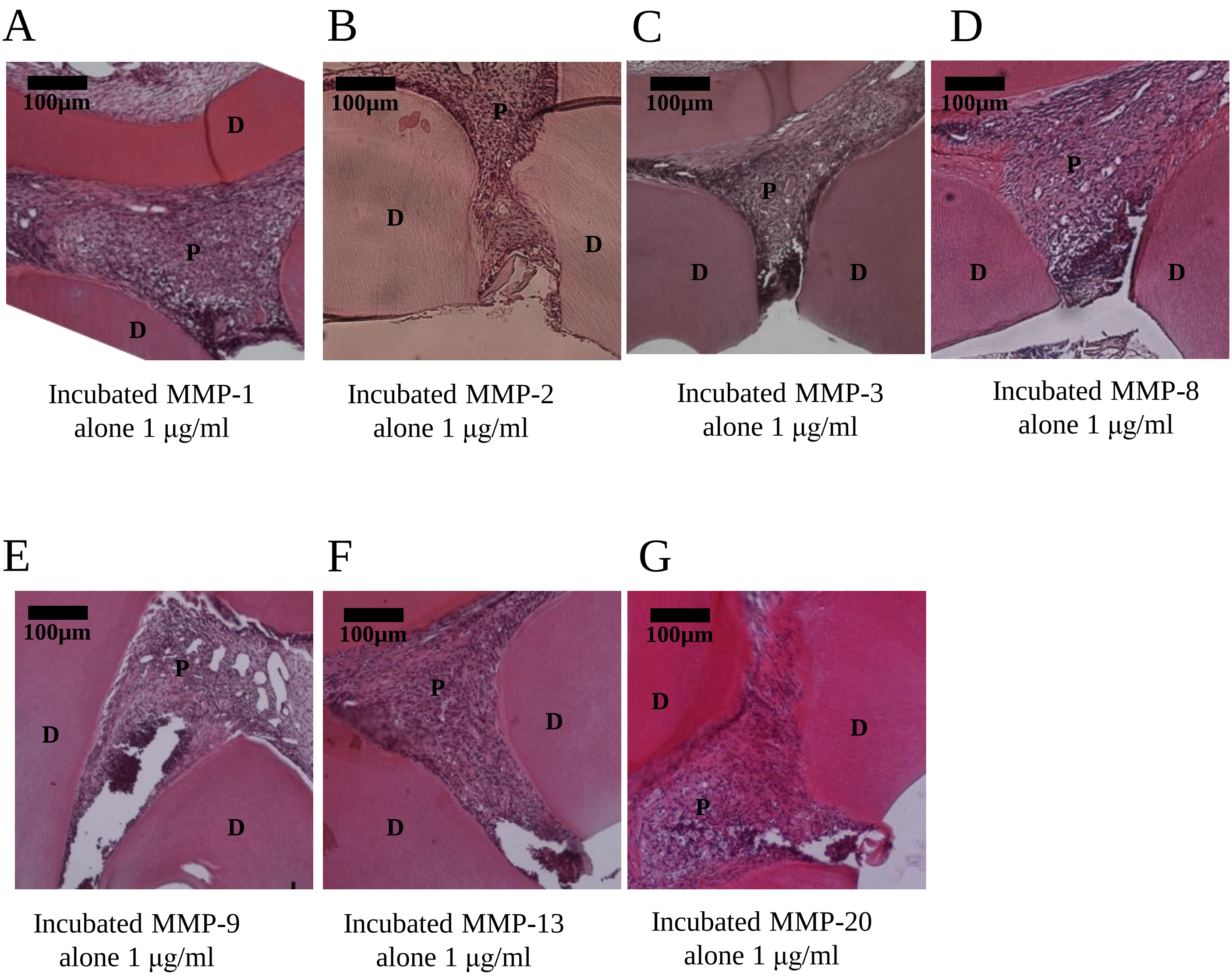

Supplementary figure 12

Histological images (100×) of tertiary dentin formation 28 days after direct pulp capping using 1 μg/ml of incubated MMP-1 (A), MMP-2 (B), MMP-3 (C), MMP-8 (D), MMP-9 (E), MMP-13 (F), and MMP-20 (G).

A

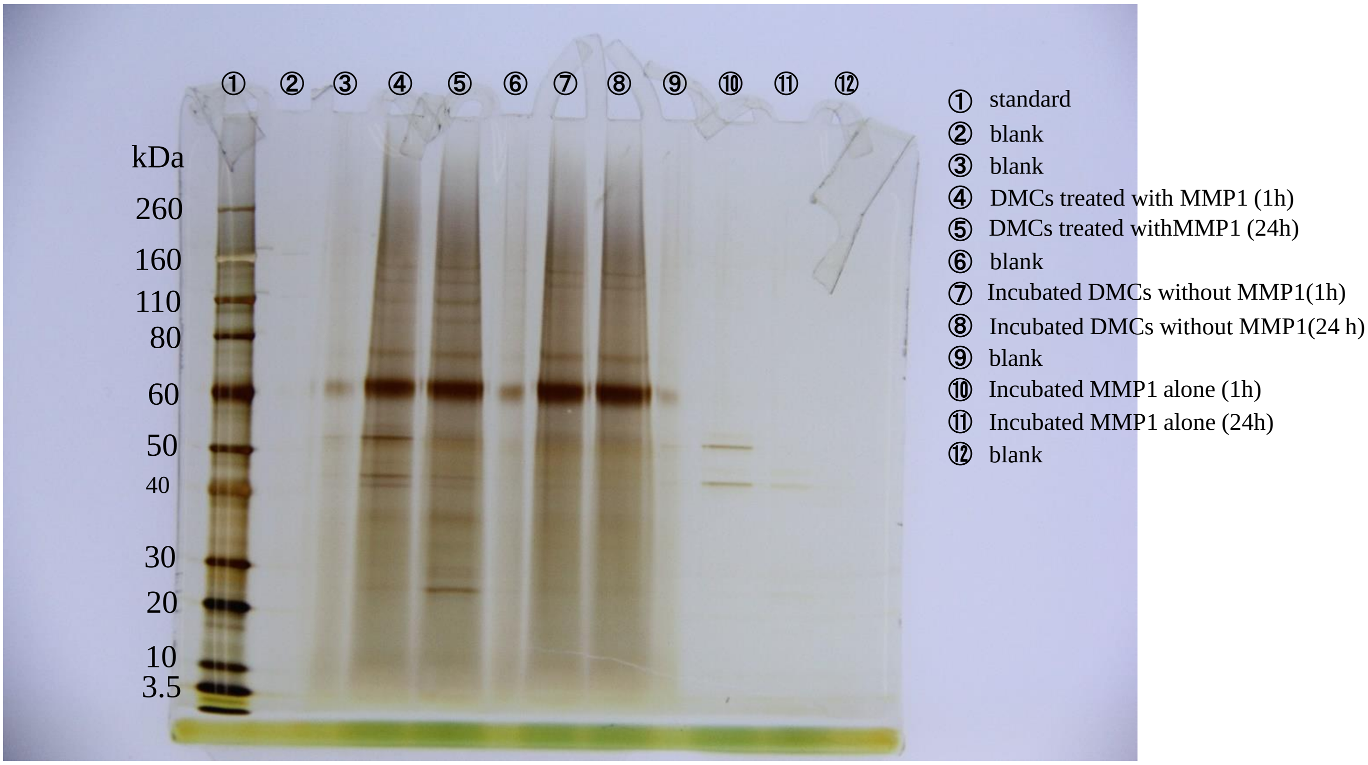

B

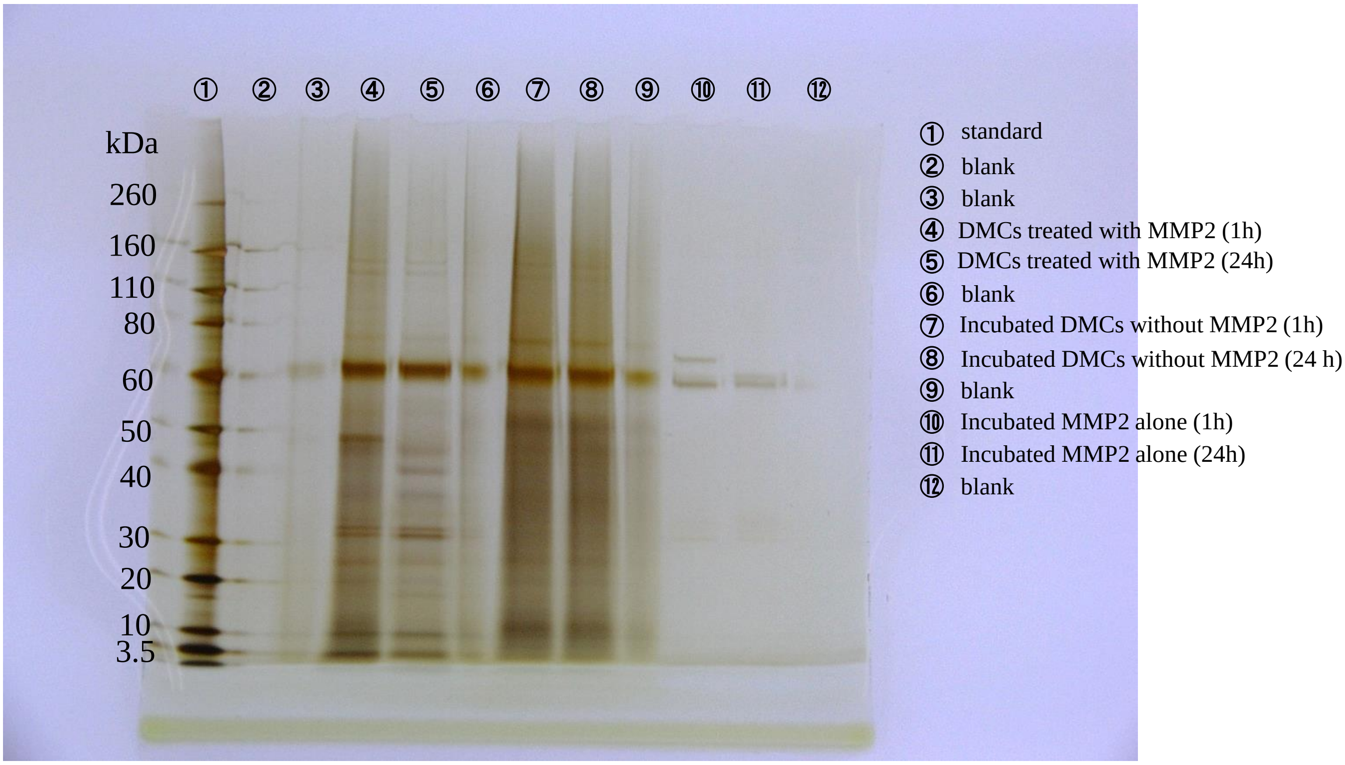

Supplementary figure 13

Protein profiles (silver staining) of DMCs treated with MMP-1 (A) or MMP-2 (B). The incubation time influenced the protein profile..

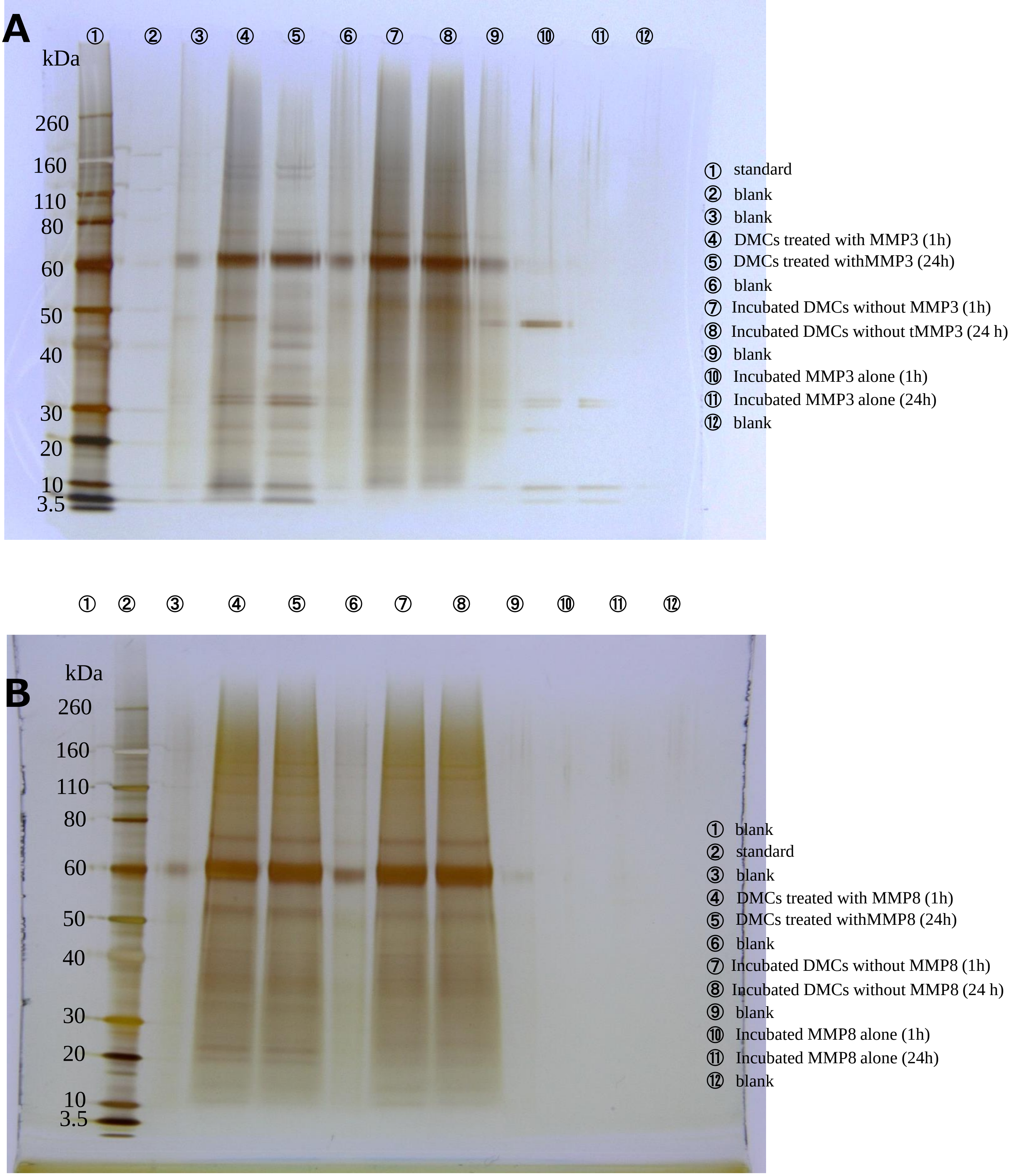

Supplementary figure 14

Protein profiles (silver staining) of DMCs treated with MMP-3 (A) or MMP-8 (B). The incubation time influenced the protein profile.

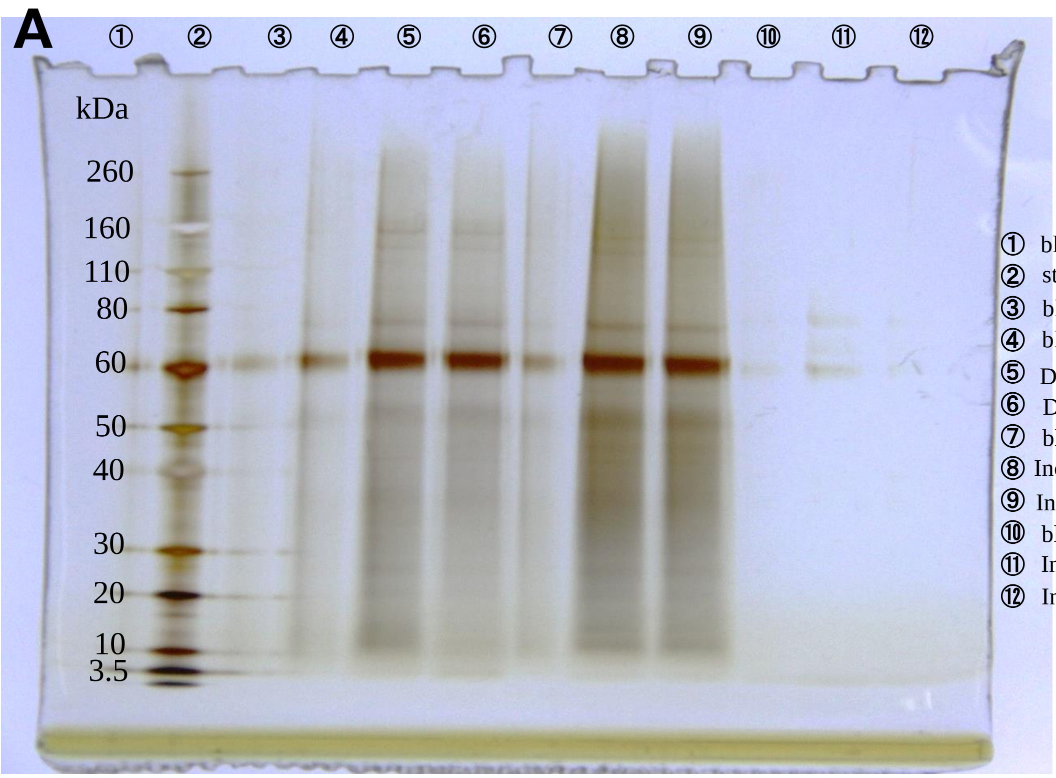

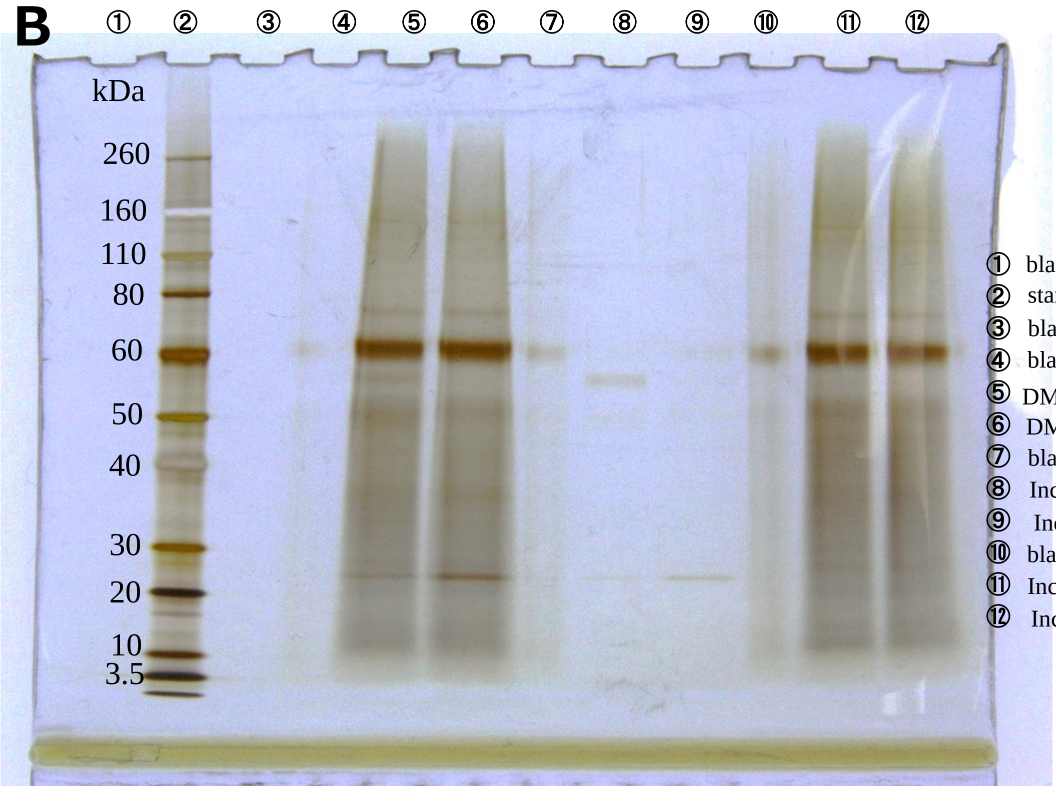

Supplementary figure 15

Protein profiles (silver staining) of DMCs treated with MMP-9 (A) or MMP-13 (B). The incubation time influenced the protein profile.

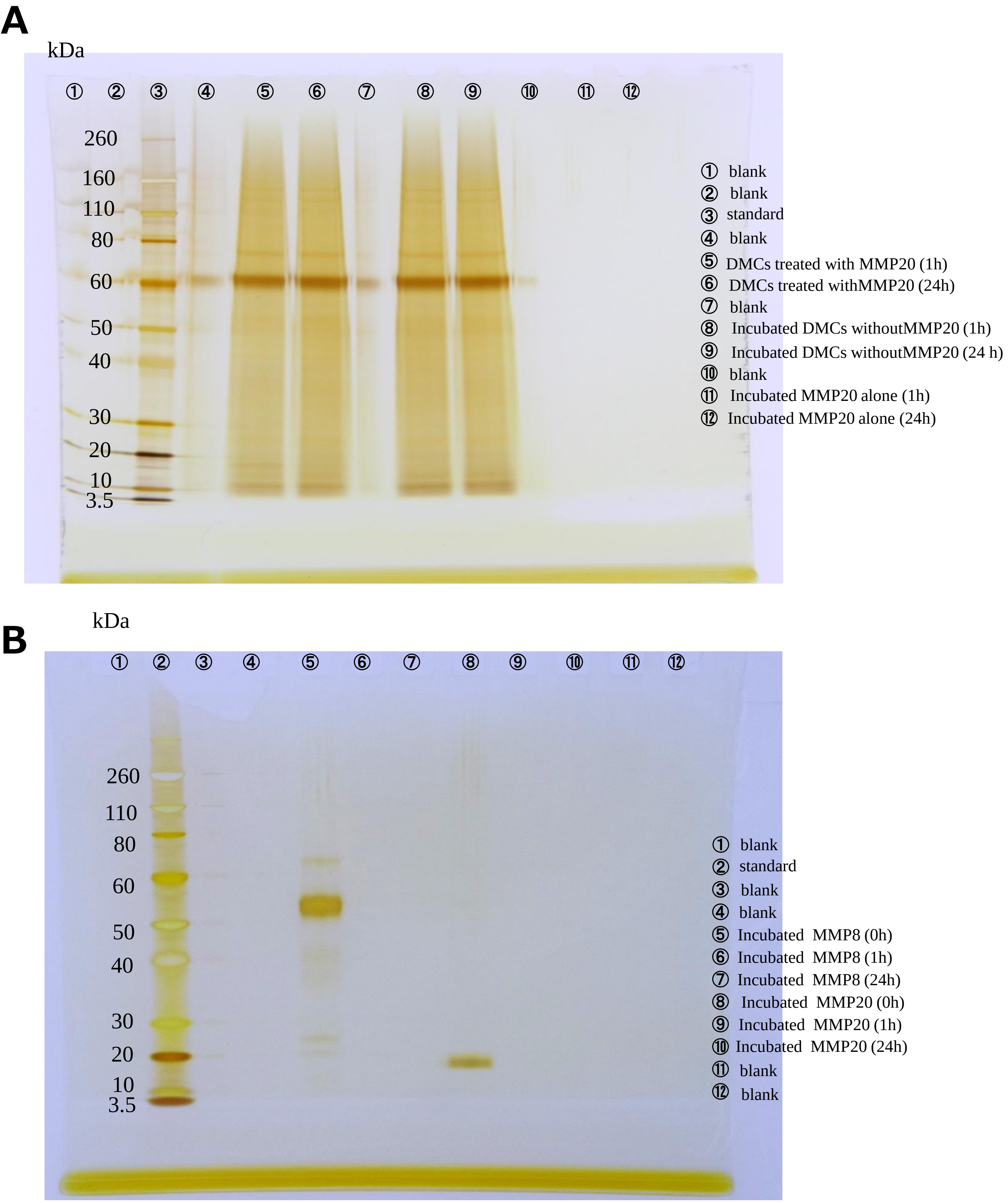

Supplementary figure 16

Protein profiles (silver staining) of DMCs treated with MMP-20 (A) or with MMP8 and MMP20 (B). MMP incubation time (0, 1, and 24 h) influenced the protein profile.
